# Spatially resolved molecular signatures of Lewy body dementia

**DOI:** 10.1007/s00401-026-02981-z

**Published:** 2026-01-26

**Authors:** Yunjung Jin, Kai Chen, Alexander Q. Wixom, Zonghua Li, Shunsuke Koga, Hiroaki Sekiya, Gisela Xhafkollari, Monica Castanedes-Casey, Hannah Santhakumar, Axel D. Meneses, Abigail J. Neff, Guojun Bu, Michael G. Heckman, Yuanhang Liu, Owen A. Ross, Dennis W. Dickson, Na Zhao

**Affiliations:** 1 Department of Neuroscience, Mayo Clinic, 4500 San Pablo Road, Jacksonville, FL 32224 USA; 2 Department of Quantitative Health Sciences, Mayo Clinic, Rochester, MN 55905 USA; 3 Present Address: Department of Pathology and Laboratory Medicine, Hospital of the University of Pennsylvania, Philadelphia, PA 19104 USA; 4 Present Address: Division of Life Science, The Hong Kong University of Science and Technology, Clear Water Bay, Hong Kong, China

**Keywords:** Lewy body dementia, *SNCA*, *APOE*, Spatial transcriptomics, Reelin

## Abstract

Lewy body dementia (LBD), encompassing dementia with Lewy bodies and Parkinson’s disease dementia, is neuropathologically defined by neuronal accumulation of α-synuclein encoded by the *SNCA* gene. Genetic risk factors strongly influence LBD susceptibility, including *SNCA* multiplication, particularly triplication, and the apolipoprotein E ε4 allele (*APOE4*), the strongest common genetic risk factor for LBD. While *SNCA* is predominantly expressed in neurons and *APOE* primarily in glial cells, how these genetic factors converge to impact neuronal vulnerability and regional pathology in the human brain remains poorly understood. Here, we applied spatial transcriptomics to postmortem temporal cortex tissue from LBD cases with *SNCA* triplication or different *APOE* genotypes, alongside age- and sex-matched controls, to map gene expression within intact cortical architecture. We identified layer 5 of the gray matter as a particularly vulnerable region, characterized by elevated *SNCA* expression, pronounced synaptic and metabolic dysregulation, and exacerbation of these alterations in *APOE4* carriers. Reelin signaling emerged as a core Lewy body-associated pathway disrupted across cortical layers, validated in independent postmortem cohorts and human-induced pluripotent stem cell (iPSC)-derived cortical organoids. In contrast, white matter exhibited distinct molecular alterations, including disrupted myelination pathways, with *APOE4* carriers showing increased myelin debris and glial responses compared with non-carriers. Cell-type deconvolution informed by single-nucleus RNA sequencing further revealed *APOE4*-associated impairments in neuronal vulnerability and intercellular communication. Together, these findings define spatially and cell-type-specific mechanisms through which *SNCA* dosage and *APOE4* genotype impact LBD pathology, providing insight into regionally distinct disease processes and potential targets for genetically stratified therapeutic interventions.

**Supplementary Information:**

The online version contains supplementary material available at 10.1007/s00401-026-02981-z.

## Background

Lewy body dementia (LBD), including dementia with Lewy bodies (DLB) and Parkinson’s disease (PD) dementia (PDD), is one of the major forms of Alzheimer’s disease (AD)-related dementias [2, 15, 27]. LBD is characterized by the presence of Lewy bodies and Lewy neurites containing aggregated α-synuclein (α-SYN) protein, encoded by the *SNCA* gene [17, 31, 34, 44]. However, the mechanisms underlying the neuronal vulnerability to α-SYN aggregation and toxicity in LBD remain poorly understood.

It is well-established that *SNCA* multiplication—particularly triplication—leads to PD and LBD [8, 9, 18, 22, 36, 38, 40, 48], suggesting that the level of *SNCA* expression in neurons may influence susceptibility to Lewy pathology. Although *SNCA* triplications are very rare [5], their pronounced clinical features and rapid dose-dependent disease progression make them a valuable resource for understanding LBD pathogenesis. We previously demonstrated disease-related phenotypes in human-induced pluripotent stem cell (iPSC)-derived cortical organoids and excitatory neurons from patients with *SNCA* triplication, including α-SYN aggregation as well as synaptic and bioenergetic dysfunction [24]. These findings indicate that *SNCA* triplication alone can disrupt normal neuronal function in vitro. However, how *SNCA* expression contributes to neuronal vulnerability in human brains in the context of triplication, and whether this mechanism is also relevant to LBD cases without *SNCA* triplication, remains unclear.

In addition, the apolipoprotein E ε4 allele (*APOE4*) has emerged as the most significant genetic risk factor for LBD, as identified by both genome-wide association studies (GWAS) and large-scale whole-genome sequencing (WGS) studies [13, 20], similar to its well-established association with AD [28]. Our group and others have previously shown that *APOE4* can exacerbate α-SYN pathology independently of classical AD pathologies [14, 16, 25, 43, 50], although LBD brains frequently exhibit coexisting amyloid pathology [44]. In the brain, *APOE4* is primarily expressed by glial cells, such as astrocytes, and by microglia under disease conditions, and has been implicated in various pathogenic mechanisms, such as impaired lipid and cholesterol metabolism, neuroinflammation, and disruption of glial homeostasis [46, 51]. In contrast, *SNCA* is predominantly expressed in neurons [24]. Thus, how *APOE* genotype influences α-SYN-related Lewy pathology within neurons, and contributes to neuronal vulnerability, remains poorly understood.

To address these gaps, we analyzed postmortem human brains from LBD cases, including those with *SNCA* triplication and different *APOE* genotypes, along with age- and sex-matched controls. We performed spatial transcriptomics on tissue from the temporal cortex, allowing for high-resolution mapping of gene expression within intact tissue architecture [7, 10, 41, 45]. We identified layer-specific *SNCA* expression patterns aligned with Lewy body distribution, along with synaptic and metabolic dysregulation and impaired Reelin signaling in gray matter and disrupted myelination pathways in white matter. Using cell-type deconvolution informed by single-nucleus RNA sequencing (snRNA-seq) data from the same cases [24], we further uncovered *APOE4*-associated impairments in neuronal vulnerability and cell–cell communication that may contribute to increased LBD risk. Overall, our findings provide new insights into how *SNCA* and *APOE4* shape the spatial and cellular molecular landscape of LBD pathology.

## Materials and methods

### Human postmortem brain samples

This study was conducted in accordance with a protocol approved by the Mayo Clinic Institutional Review Board (no. 15-009452). A total of four control cases and six LBD cases were used for spatial transcriptomics; a subset of these control and LBD cases had previously undergone snRNA-seq analysis [24] (Supplementary Table 1). For western blot validation, 16 control cases and 16 LBD cases were included (Supplementary Table 6). In addition, expanded cohorts including 19–23 and 13–16 LBD cases per *APOE* genotype were analyzed for western blotting (Supplementary Table 8) and immunostaining validation (Supplementary Table 11), respectively. All cases were obtained from the Brain Bank for Neurodegenerative Disorders at Mayo Clinic, Jacksonville. Each case underwent standardized neuropathologic sampling and evaluation as previously described [16, 26, 39]. Briefly, thioflavin-S fluorescence microscopy was used to assess AD neuropathologic change, including Braak neurofibrillary tangle stage and Thal amyloid phase [6, 42]. Lewy pathology was evaluated using α-SYN immunohistochemistry, with Lewy bodies quantified in the cingulate, inferior parietal, middle frontal, parahippocampal, and superior temporal gyri, as well as in the substantia nigra [16, 34].

### 10× Visium spatial transcriptomics of human brain

#### Library preparation and sequencing

Formalin-fixed paraffin-embedded (FFPE) brain sections were deparaffinized, stained with H&E, and decrosslinked according to the Visium CytAssist Spatial Gene Expression for FFPE protocol (CG000520 rev. B, 10× Genomics). H&E-stained tissues were imaged at 20X magnification using the Aperio AT2 digital imaging scanner (Leica Biosystems, Germany). Immediately after decrosslinking, libraries were prepared according to the user’s guide for Visium CytAssist Spatial Gene Expression Reagent Kits (CG000495, rev. E, 10× Genomics). Final libraries were sequenced using the Illumina HiSeq 4000.

#### Spatial transcriptomics data analysis

Briefly, sequencing data were processed and quantified utilizing the 10X Genomics Space Ranger Software Suite (version 3.1.0) using the 10× human reference (GRCh38-2.1.0.pre-mrna, https://support.10xgenomics.com/single-cell-gene-expression/software/pipelines/latest/advanced/references). The 10× Loupe Browser Cytassist framework was utilized to establish tissue boundaries for each sample and identify spot locations to quantify gene counts. The resulting gene count matrices were imported into R (v.4.2.2) and the Seurat package (version 5.0.1) for subsequent analysis [21]. Spots with gene counts below 500 or mitochondrial genes over 40% were excluded from the downstream analysis.

#### Spot clustering and annotation

Spot integration and clustering were performed using the Harmony package (v1.0) implemented in Seurat (v5.0.1) following the standardized SCT v2 workflow. The top 3000 highly variable genes identified by variance-stabilizing transformation were used for downstream analyses. Percent mitochondrial content (percent.mt) and spot cell-cycle phase were regressed out during data scaling, and the top 30 principal components (PCs) were used for clustering with the RunHarmony function. Dimensionality reduction was performed using UMAP, t-SNE, and PCA, and nearest neighbors were identified according to Seurat recommendations. Clustering was performed across multiple resolutions and assessed for stability using clustree [49], with a final resolution of 0.6 selected. Gray matter (GM) layers, white matter (WM), and Lewy body (LB) annotations generated by neuropathologists were imported for each sample using the Loupe Browser. Clusters were manually curated and assigned to cortical layers based on the expression of known marker genes and their overlap with neuropathologist annotations, which were given higher priority during cluster assignment. These annotations enabled stratification of multiple groups for downstream statistical analyses. The Loupe Browser was additionally used to annotate LB-positive (LB+) spots and LB-surrounding (LBsur) spots proximal to LBs identified by neuropathologists, resulting in the identification of 3 LB+ spots and 9 LBsur spots. LB+ spot counts were aggregated by cortical layer and sample. Paired *t *tests were used to assess differences in LB+ spot occurrence across layers.

#### Spot deconvolution

SCT-normalized gene expression profiles from each spot were deconvoluted using spacexr (v.2.2.0) [7]. Our previously published snRNA-seq data [24] from an overlapping patient cohort was used as the deconvolution reference following spacexr recommendations. Cell-type weights were isolated and utilized as an analog for cell-type prevalence within each spot based on spacexr guidelines. Cell-type expression profiles per spot were isolated from the deconvolution object, and all metadata were merged to the original Seurat object to allow for visualization and further statistical analysis. Briefly, deconvoluted cell populations were aggregated based on layer, sample, and sample *APOE* genotype.

#### Differential gene expression analysis

The differentially expressed gene (DEG) analysis was completed for LBD vs control for all layers, *APOE* genotypes, *SNCA* expression levels, brain matter types, deconvoluted cell types (including excitatory neurons, inhibitory neurons, astrocytes, oligodendrocytes, OPCs, microglia, and vascular cells), and LB-annotated and LB-surrounding spots using Seurats’ FindMarkers function testing for significance via Wilcoxon rank-sum test for our spot-based analysis. Subsets of spots were summarily aggregated per condition to perform a pseudo-bulk DEG analysis using FindMarkers and DESeq2 to test for significance. *p *values were corrected by Bonferroni correction as suggested by Seurat. Genes with spot-based |logFC| ≥ 0.25, consistent direction of expression change in spot and pseudo-bulk based logFC, FDR < 0.05 in the spot-based analysis, and *p* < 0.05 in the pseudo-bulk analysis were defined as our DEGs. DEGs were visualized using upset plots using UpSetR package to identify consistent changes across different conditions.

#### IPA pathway analysis

IPA pathway analysis for each comparison was performed using DEGs. IPA pathways, found to be enriched in specific *APOE* genotypes in the excitatory neurons in LB+, LB-surrounding spots, and/or LB− spots, were visualized via a dot plot using ggplot2 (v3.5.1).

#### Cell to cell communication

CellChat (v.2.1.0) [23] was utilized to determine differential communication occurring when subsetting samples based on their *APOE* genotype. We followed the package recommendations for spatial analyses. Briefly, CellChat objects were created for each subset of samples, overexpressing genes and interactions were identified. Communication and pathway probabilities were calculated and filtered. Networks were aggregated, and centrality was computed before subsetting and merging to create the CellChat object. Visualizations were generated using the built-in functions from CellChat.

### Cerebral organoid culture

To validate the key findings, cerebral organoids were differentiated from a set of isogenic iPSC lines purchased from ATCC, including one parental *SNCA*-triplication iPSC line and an isogenic control iPSC line with a normal copy of the *SNCA* genes, as previously described [24, 47]. STEMdiff™ Cerebral Organoid Kit (Stemcell Technologies) was used to generate cerebral organoids following manufacturer’s instructions as we previously reported [24]. Briefly, on Day 0, iPSC colonies were dissociated into a single cell suspension with Accutase, where 15,000 cells were seeded into a U-bottom ultra-low-attachment 96-well plate in EB formation media (medium A) supplemented with 10 μM Y-27632. On Day 2 and Day 4, an additional 100 μl of medium A was added per well. On Day 5, EBs were moved to 48-well low-attachment plates in neural induction medium (medium B) and left for an additional 3–5 days. EBs were further embedded into 20 μl of Matrigel and cultured in neural expansion medium (medium C + D) for 3 days in six-well low-attachment plates for organoid formation. Finally, organoids were transferred to 10 cm dishes and moved to an orbital shaker for further culture in neural culture medium (medium E). After 4 weeks, medium E was replaced with neuronal maturation medium consisting of: DMEM/F12 + Neurobasal Medium (1:1) supplemented with N2, B27, BDNF (20 ng/ml), GDNF (20 ng/ml), ascorbic acid (200 μM) and dbcAMP (100 nM) (Sigma Aldrich). Cerebral organoids were harvested after 2 months of differentiation, and 3–4 organoids were pooled for biochemical analysis.

### Protein extraction from frozen human brain tissue and organoids

Human brain tissue from the superior temporal cortex and cerebral organoids were subjected to two-step sequential protein extractions using Tris-buffered saline (TBS) buffer and detergent-containing buffer (TBSX, TBS with 1% Triton X-100) to obtain the buffer-soluble (TBS) and detergent-soluble (TBSX) proteins, respectively. Briefly, organoids and brains were homogenized in ice-cold TBS buffer containing a protease inhibitor cocktail (Roche) and a phosphatase inhibitor (Roche), sonicated, and incubated at 4 °C for 30 min with end-over-end agitation. The supernatant was collected after centrifugation at 100,000×*g* for 30 min for organoids and 60 min for brains at 4 °C as TBS fraction. The residual pellet was re-homogenized in TBSX buffer with protease and phosphatase inhibitors, sonicated, incubated at 4 °C for 30 min for organoids and 60 min for brains with end-over-end agitation, and centrifuged as above to obtain the supernatant as the TBSX fraction for further assays.

### Western blotting

The proteins were resolved by sodium dodecyl sulfate polyacrylamide gel electrophoresis (SDS-PAGE), and transferred to polyvinylidene difluoride membranes, which were subsequently blocked using 5% milk in PBS. After blocking, proteins were detected with a primary antibody overnight at 4 °C. The next day, membranes were washed, and probed with horseradish peroxide (HRP)-conjugated secondary antibody and developed with enhanced chemiluminescence imaging. The primary antibodies were as follows: Reelin (Abcam, 1:1000; Santa Cruz, 1:1000), ApoER2 (Abcam, 1:1000), Phospho-Dab1 (Abcam, 1:1000), Dab1 (Abcam, 1:1000), Tuj1 (Sigma Aldrich, 1:5000), and β-actin (Sigma Aldrich, 1:5000).

### Immunofluorescence

FFPE brain sections from the temporal cortex were used for immunofluorescent staining. After deparaffinization and rehydration, antigen retrieval was performed by heating the sections in antigen retrieval solution (Target Retrieval Solution, Citrate pH 6; Dako) for 30 min. Subsequently, the slides were blocked with serum-free protein block (Agilent Technologies) and incubated with primary antibodies overnight at 4 °C. The primary antibodies were as follows: phospho-α-synuclein (Abcam, 1:300), Iba1 (Abcam, 1: 300), GFAP (Millipore Sigma, 1:400), MBP (Millipore Sigma, 1:300), dMBP (Millipore, 1:250), Olig2 (R&D system, 1:100), and CD68 (Dako, 1:200). After primary antibody incubation, the slides were incubated with secondary antibodies targeting the respective host species for 2 h at room temperature. To quench autofluorescence, all slides were treated with 1% Sudan Black B. Whole brains were imaged using Akoya PhenoImager at 20X magnification. Images were annotated by GM and WM and analyzed by QuPath. For each detected LB, the centroid and minimum enclosing circle radius were determined using OpenCV (v4.12.0). To analyze LB-associated Iba1 and GFAP signals, concentric annular masks were generated by extending the LB radius by 100 and 150 μm. LB-related image processing and spatial analyses were performed using Python (v3.12). The investigators were blinded to the demographic information of the slides throughout the process of staining, imaging, and image analysis.

### Statistical analysis

All data were reported as mean values ± SEM unless otherwise indicated. Group comparisons were performed using either the Student’s *t* test or the Wilcoxon rank-sum test with Bonferroni correction, as indicated in the Methods and Figure legends. Statistical analyses for biochemical assays were performed using GraphPad Prism v8.4.3 (GraphPad Software). All statistical tests were two-sided. Details regarding the statistical tests used, sample sizes, and significance thresholds for each analysis are provided in the figure legends.

## Results

### Layer-specific molecular alterations in the temporal cortex of LBD

To understand the molecular phenotypes of LBD at the spatial level, we utilized spatial transcriptomics to analyze gene expression signatures in the temporal cortex, one of the brain regions most vulnerable to LBD [37]. To account for genetic risk factors such as *APOE* and *SNCA* [13], we included four autopsy-confirmed sporadic LBD cases and four age-matched control cases, with an equal distribution of *APOE3* and *APOE4* genotypes (Supplementary Table 1). In addition, we included two early-onset LBD cases with *SNCA* gene triplication (referred to as LBD (SNCA Tri)), one carrying the *APOE3* genotype and the other *APOE4* (Supplementary Table 1). All cases had minimal AD co-pathology to reduce confounding effects.

**Fig. 1:**
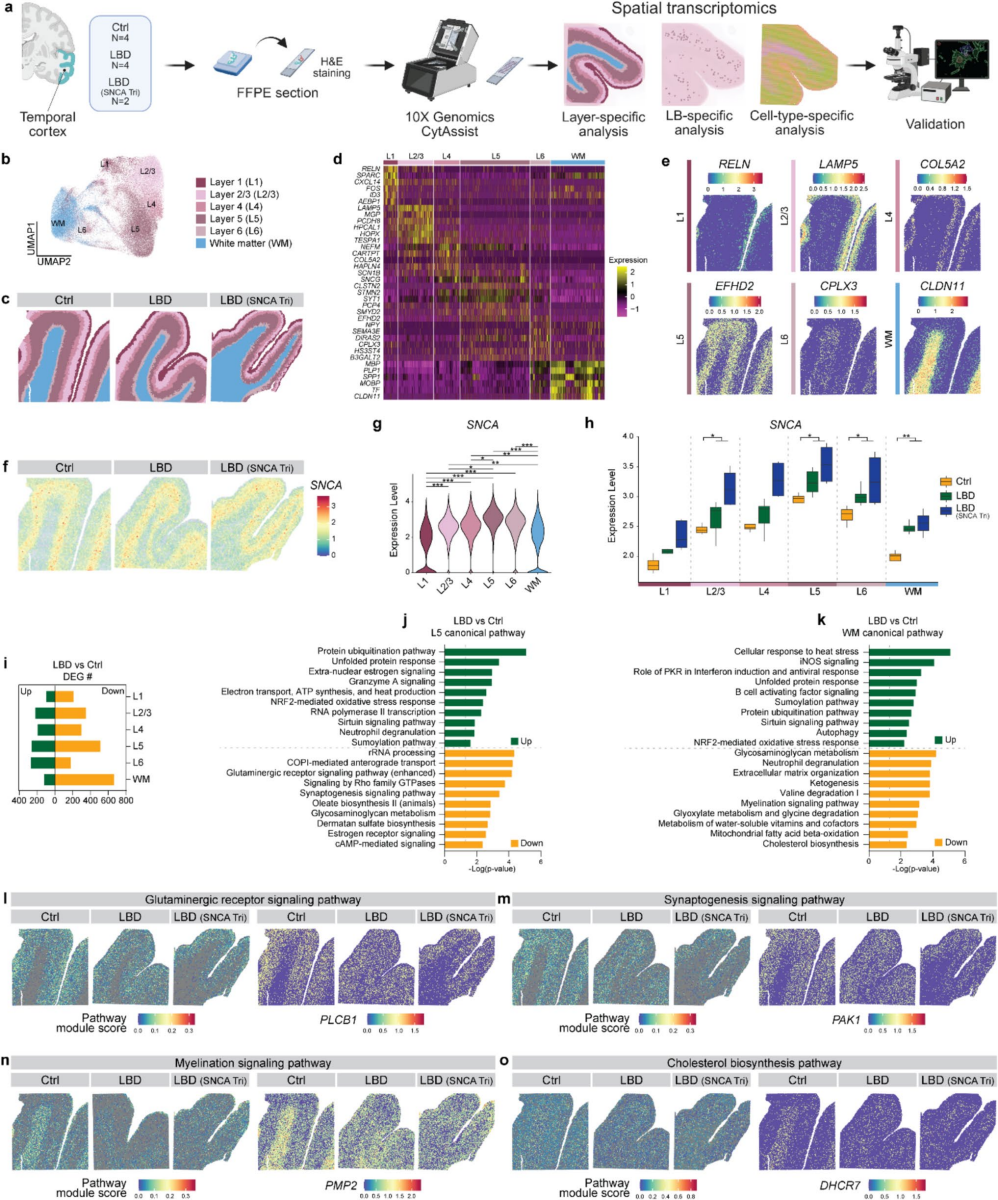
Spatially resolved transcriptional signatures reveal differences between LBD and control brains. **a** Graphical overview of spatial transcriptomics in control (Ctrl) and LBD brains, including LBD cases with *SNCA* Triplication (LBD (SNCA Tri)). UMAP (**b**) and spatial plots (**c**) showing the annotation of gray matter (GM) layers (Layer 1 (L1), Layer 2/3 (L2/3), Layer 4 (L4), Layer 5 (L5), Layer 6 (L6)) and the white matter (WM) in Ctrl and LBD brains. Heatmap of marker gene expression across GM layers and WM (**d**) and representative spatial plots of marker genes for each GM layer and WM (**e**). **f** Spatial gene expression plots of *SNCA* in Ctrl and LBD brains. **g** Violin plot showing *SNCA* gene expression across GM layers and WM. Statistical significance was assessed using the Wilcoxon rank-sum test with Bonferroni correction. *p < 0.05; **p < 0.01; ***p < 0.001. **h** Box plot showing *SNCA* expression grouped by disease status. Statistical analyses were performed using Student’s *t* test. *p < 0.05; **p < 0.01. **i** Bar graph showing the number of differentially expressed genes (DEGs) comparing LBD and control brains across GM layers (L1–L6) and WM. IPA canonical pathway analysis of DEGs enriched in L5 (**j**) and WM (**k**) comparing LBD and Ctrl brains. Selected downregulated pathways from L5 (**l**, **m**) and WM (**n**, **o**), with spatial plots showing the pathway module score (*left*) and representative DEG expression in the pathways (*right*). Ctrl cases, *N* = 4; LBD cases, *N* = 6, including LBD (*SNCA* Triplication) cases with equal *APOE* genotype distribution

FFPE sections of the temporal cortex from all cases were first stained with H&E to visualize anatomical structure (Supplementary Fig. 1a). Regions of interest were then transferred onto 10× Visium slides using CytAssist, followed by spatial transcriptomics analysis to characterize layer-specific (Supplementary Fig. 1b), Lewy body (LB)-specific (Supplementary Fig. 1c), and deconvoluted cell-type-specific molecular signatures in LBD, with subsequent validation (Fig. 1a; Supplementary Table 2). Spatial transcriptomics data were subjected to quality control assessment prior to downstream analyses. The median UMI counts per spot (nCount) and the median number of detected genes per spot (nFeature) were comparable between control and LBD samples (Supplementary Fig. 2a, b), indicating similar sequencing depth and transcript capture efficiency.

To identify brain anatomical structures based on molecular signatures, we first performed unsupervised clustering and dimensionality reduction of spatial transcriptomics spots without batch correction to assess biological and sample-wise variability. The unsupervised Uniform Manifold Approximation and Projection (UMAP) revealed segregation of spots by biological characteristics and sample origin (Supplementary Fig. 3a–c), indicating preserved biological heterogeneity across samples prior to integration. Subsequently, we performed unbiased clustering following Harmony-based integration to reduce sample-specific effects while retaining shared biological features. This analysis revealed clear segregation of gray matter (GM) cortical layers (L1, L2/3, L4, L5, and L6) and white matter (WM), as demonstrated by UMAP analysis (Fig. 1b; Supplementary Fig. 3e–g) and their spatial distribution in tissue sections (Fig. 1c). Layer-specific organization was further supported by the expression patterns of canonical marker genes for each GM layer and WM (Fig. 1d, e).

To investigate the spatial expression pattern of the *SNCA* gene, we mapped its expression in these spatial transcriptomic slides and found that *SNCA* expression was layer-specific (Fig. 1f). It showed the highest abundance in layer 5 (L5) in both control and LBD cases (Fig. 1g, h). Moreover, *SNCA* expression was elevated in LBD compared to controls across all cortical layers and WM, with the most significant increases observed in L5, as well as in L2/3, L6, and WM (Fig. 1h).

To investigate layer-specific molecular changes associated with LBD, we performed spot-based DEG analysis comparing LBD and control brains within each GM layer and WM (Fig. 1i; Supplementary Table 3). The greatest number of DEGs was observed in L5 and WM (Fig. 1i). In L5, DEGs were enriched in upregulated pathways such as protein ubiquitination and unfolded protein response, while pathways associated with synaptic signaling were predominantly downregulated (Fig. 1j). Similar pathway alterations were also observed across other cortical layers (Supplementary Table 4). In WM, upregulated pathways included cell stress response, protein ubiquitination, and unfolded protein response, whereas pathways involved in myelination and lipid metabolism were downregulated (Fig. 1k). To visualize these pathway-level changes, we calculated pathway module scores and overlaid both the scores and representative genes from selected pathways onto the spatial transcriptomic map. Specifically, the genes involved in the glutaminergic receptor signaling pathway (e.g., *PLCB1* gene) and the synaptogenesis signaling pathway (e.g., *PAK1* gene) were significantly reduced in LBD, particularly in L5 (Fig. 1l, m). The genes involved in the myelination signaling pathway (e.g., *PMP2* gene) and the cholesterol biosynthesis pathway (e.g., *DHCR7* gene) were significantly downregulated in WM of LBD cases compared to controls (Fig. 1n, o).

Together, these findings highlight prominent layer-specific transcriptional alterations in LBD, with *SNCA* showing high abundance in L5, and increased *SNCA* expression in LBD accompanied by dysregulation of synaptic pathways in the GM, particularly in L5, and disruption of myelination pathways in WM.

### LB-associated molecular dysregulation in LBD

**Fig. 2:**
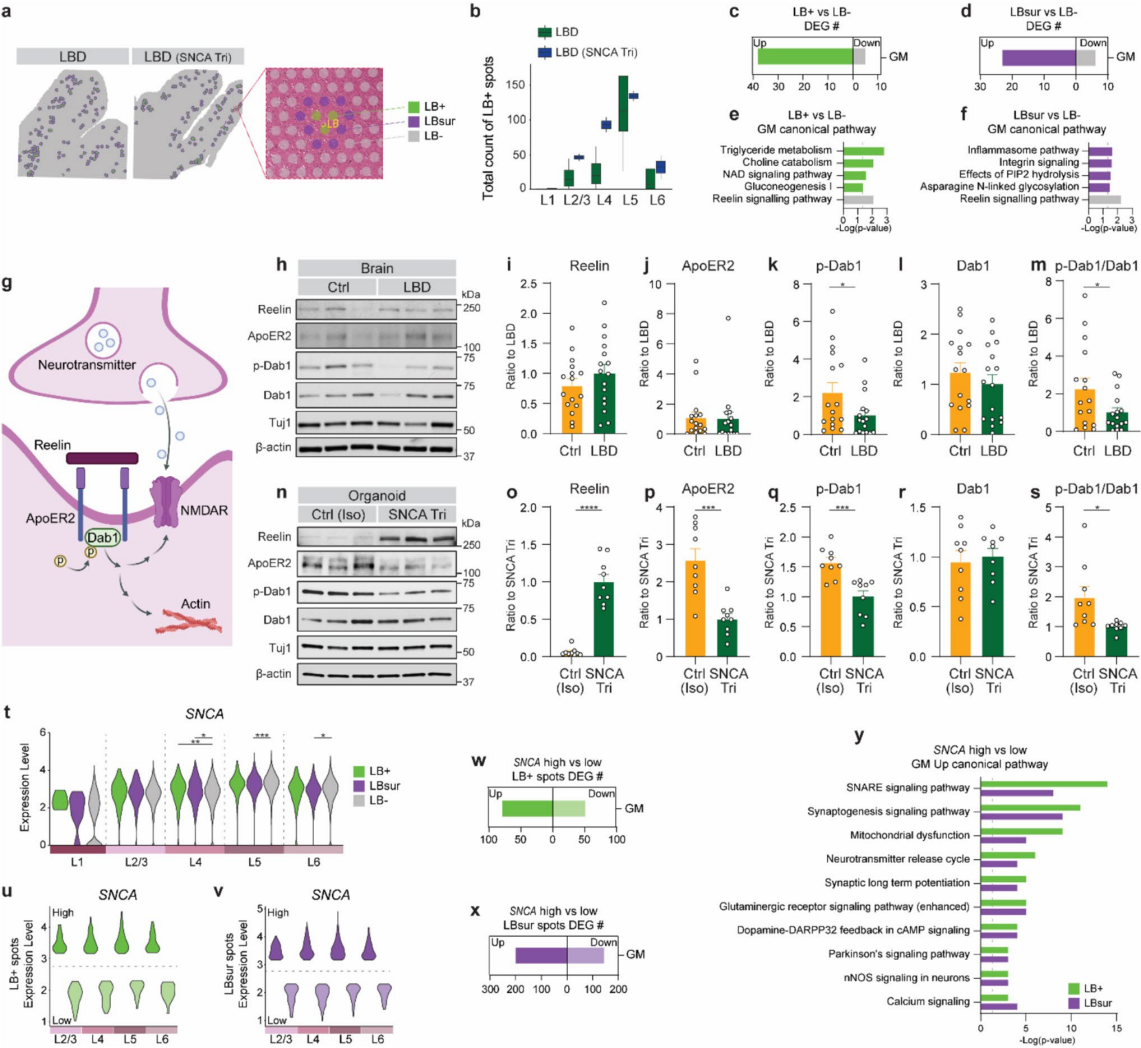
Transcriptional signatures related to LB pathology. **a** Spatial plot and H&E-stained image with annotation of LB-positive spots (LB+, *green*), LB-surrounding spots (LBsur, *purple*), and LB-negative spots (LB−, *gray*) in LBD brains. **b** Count of LB+ spots in LBD brains across GM layers (L1–L6). Statistical analyses were performed using Student’s *t* test. *p < 0.05; **p < 0.01. Bar graphs showing the number of DEGs comparing LB+ spots to LB− spots (**c**) and LBsur spots to LB− spots (**d**) in LBD brains across GM layers (L1–L6). IPA canonical pathway analysis of DEGs enriched in GM, comparing LB+ spots to LB− spots (**e**) and LBsur spots to LB− spots (**f**) in LBD brains. **g** Graphical summary of the Reelin signaling cascade in the brain. **h–s** Validation of Reelin signaling through western blotting in Ctrl and LBD human brains (**h**–**m**) and an isogenic set of control (Ctrl (Iso)) and *SNCA* triplication (SNCA Tri) cortical organoids (**n**–**s**). Representative gel images (**h**, **n**) are shown. Quantification of Reelin (**i**, **o**), ApoER2 (**j**, **p**), p-Dab1 (**k**, **q**), Dab1 (**l**, **r**) levels, and p-Dab1/Dab1 ratio (**m**, **s**) in TBSX fractions of human brains and organoids. Results were normalized to β-actin levels. Human brains: *N* = 16 samples per group; each dot represents an individual case. Isogenic organoids: experiments conducted in triplicate with three independent experiments; each dot represents an individual replicate. Data are presented as mean ± SEM. Statistical analyses were performed using Student’s *t* test. *p < 0.05; **p < 0.01; ***p < 0.001. **t** Violin plot showing *SNCA* gene expression in LB+, LBsur, and LB− spots across GM layers (L1–L6). Statistical significance was assessed using the Wilcoxon rank-sum test with Bonferroni correction. *p < 0.05; **p < 0.01; ***p < 0.001; ****p < 0.0001. Violin plots of *SNCA* expression in *SNCA*-high and *SNCA*-low expressing spots in LB+ (**u**) and LBsur (**v**) spots in LBD brains. Bar graphs show the number of DEGs comparing *SNCA*-high and *SNCA*-low expressing spots of LB+ (**w**) and LBsur (**x**) in LBD brains. **y** IPA canonical pathway analysis with upregulated DEGs comparing *SNCA*-high and *SNCA*-low expressing spots in LB+ and LBsur spots in LBD brains

To further investigate spatial transcriptomic alterations associated with LB pathology, we annotated LBs in LBD brains using H&E-stained sections. With the Loupe Browser, we classified the spatial transcriptomic spots into three categories: LB-positive (LB+) spots containing LBs, LB-surrounding (LBsur) spots adjacent to LBs, and LB-negative (LB−) spots for the remaining areas. (Fig. 2a; Supplementary Fig. 1c; Supplementary Fig. 3d, h). Overlaying these annotated spots with layer-specific definitions revealed that LB + spots were predominantly located in L5 (Fig. 2b; Supplementary Fig. 3 h), consistent with the highest *SNCA* expression observed in this layer (Fig. 1f–h). We then performed DEG and pathway analyses comparing LB+ versus LB− spots and LBsur versus LB− spots within the same layer, aiming to identify both shared and distinct transcriptional signatures associated with pathology and its immediate surroundings (Fig. 2c–f; Supplementary Table 5). Due to variability in the number and distribution of LBs across layers and samples, layer-specific comparisons were limited. Therefore, we combined LB+ spots across all layers within the GM for DEG analysis. This revealed that LB+ spots exhibited upregulation of bioenergetic metabolism pathways (Fig. 2e), while LBsur spots were enriched for upregulation of immune-related pathways (Fig. 2f). Notably, the Reelin signaling pathway was consistently downregulated in both LB+ and LBsur spots compared to LB− spots in the GM (Fig. 2e, f). This observation is consistent with our previous snRNA-seq study, where Reelin signaling was dysregulated across multiple cell types in LBD brains compared to the controls [24], highlighting its potential role in LBD pathogenesis.

Reelin signaling has been implicated in various neurological disorders [1], with its disruption leading to impaired neurotransmission and compromised neuronal scaffold maintenance. Upon Reelin binding to its receptor ApoER2, the adaptor protein Dab1 is recruited and phosphorylated, activating its downstream pathways that regulate actin cytoskeleton dynamics or NMDA receptor (NMDAR)-mediated neurotransmission (Fig. 2g). To further validate the alterations in Reelin signaling in LBD, we assessed protein levels involved in this pathway in an independent cohort of postmortem LBD and control brains (Fig. 2h–m; Supplementary Table 6), as well as in our previously established iPSC-derived cortical organoid models from *SNCA* triplication (SNCA Tri) and isogenic controls (Ctrl (Iso)) [24] (Fig. 2n–s). In postmortem human brains, Reelin and ApoER2 protein levels did not differ significantly between LBD and control groups (Fig. 2h–j). However, phosphorylated Dab1 (p-Dab1) levels and the p-Dab1 to total Dab1 ratio were significantly decreased in LBD brains (Fig. 2h, k–m). In cortical organoids, *SNCA* Tri organoids showed increased Reelin levels (Fig. 2n, o), but reduced ApoER2 expression (Fig. 2n, p), along with decreased p-Dab1 levels and a lower p-Dab1 to total Dab1 ratio compared to isogenic controls (Fig. 2n, q–s). Together, these findings suggest that downstream Reelin signaling is impaired in LBD pathogenesis, both in postmortem human brain tissue and in cortical organoid models. The discrepancies in Reelin and ApoER2 levels between human brains and organoids may reflect differences in cell composition and disease stage, with human brains being more complex and representing end-stage pathology, while organoids may model earlier stages of disease development in neurons and astrocytes.

To determine whether *SNCA* expression levels contribute to LB pathogenesis, we compared *SNCA* levels among LB+, LBsur, and LB− spots in LBD brains. Across the GM layers, *SNCA* levels were similar between LB+ and LB− groups, except in L4, where levels differed between LBsur and LB− spots in L4, L5, and L6 (Fig. 2t). To further investigate gene expression and pathway changes associated with *SNCA* expression in these pathological regions, we stratified LB+ and LBsur spots into *SNCA*-high (top 25th percentile) and *SNCA*-low (bottom 25th percentile) subgroups (Fig. 2u, v) and performed DEG analysis between these subpopulations (Fig. 2w, x; Supplementary Table 7). *SNCA*-high spots showed enrichment in synaptic pathways, bioenergetic pathways, and Parkinson’s disease signaling in both LB+ and LBsur spots (Fig. 2y; Supplementary Table 7), aligning with the molecular changes observed when comparing LBD and control samples.

Altogether, these results indicate that both Reelin signaling disruption and *SNCA*-related perturbations in synaptic and bioenergetic pathways may contribute to LB formation in LBD.

### *APOE4*-associated increases in *SNCA* expression and dysregulation of synaptic and bioenergetic pathways in LBD

Given the critical role of *APOE4* as the strongest genetic risk factor for LBD [13], and the previously observed layer- and pathology-associated molecular dysregulation in LBD, we next examined whether *APOE4* further exacerbates molecular dysfunction in LBD. Because Reelin signaling emerged as a core LB-associated pathway in our spatial transcriptomic and validation analyses (Fig. 2e–s), we first assessed whether *APOE4* further modulates Reelin pathway components using an expanded cohort of LBD cases comprising age- and sex-matched *APOE4* carriers (E4) and *APOE3/3* (E3) individuals (Supplementary Table 8). Protein levels of Reelin, ApoER2, p-Dab1, and total Dab1 did not differ significantly between E3 and E4 within these LBD brains (Supplementary Fig. 4a–f). These results suggest that *APOE4* does not further exacerbate Reelin pathway alterations in LBD.

**Fig. 3:**
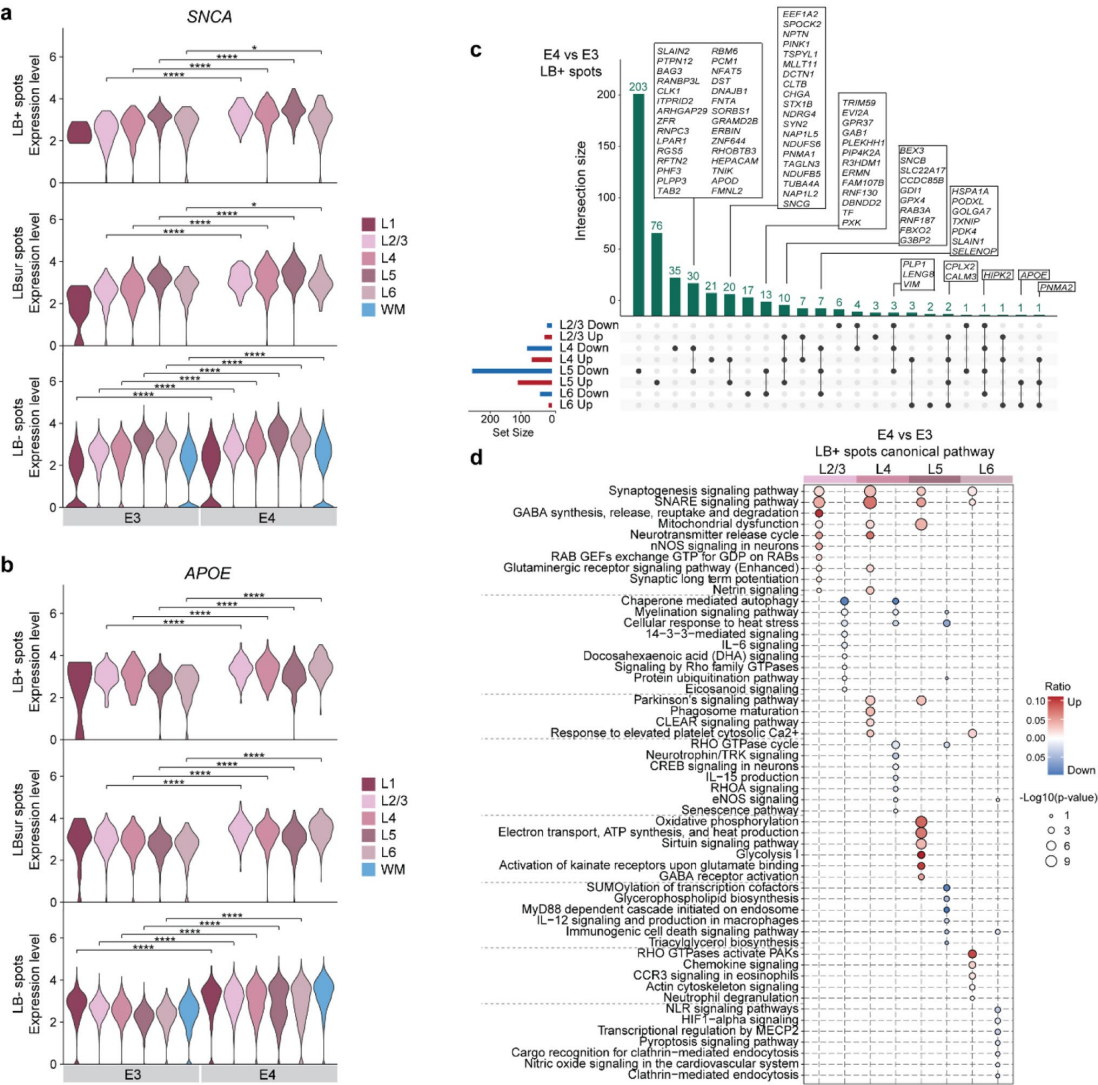
*APOE4* modulates the transcriptional signatures related to LB pathology. Violin plots of *SNCA* (**a**) and *APOE* gene (**b**) expression in LB+ spots, LBsur spots, and LB− spots in *APOE3* (E3) and *APOE4* (E4) LBD brains across GM layers (L1–L6) and WM. Statistical significance was assessed using the Wilcoxon rank-sum test with Bonferroni correction. *p < 0.05; **p < 0.01; ***p < 0.001; ****p < 0.0001. **c** Upset plot displaying DEGs in LB+ spots across GM layers (L2/3–L6) comparing E4 and E3 LBD brains. **d** Dot plot of IPA canonical pathway analysis with DEGs in LB+ spots across GM layers (L2/3–L6) comparing E4 and E3 LBD brains

We next assessed the expression levels of *SNCA* and *APOE* across LB+, LBsur, and LB− spots, and observed significantly higher expression of both genes in E4 compared to E3 within LBD brains, regardless of LB classification (Fig. 3a, b). These findings suggest that *APOE4* may contribute to LBD pathogenesis, in part, by promoting elevated *SNCA* expression. To further explore *APOE4*-related molecular changes within LB+ spots, we performed DEG analysis comparing E4 versus E3 LBD brains (Fig. 3c; Supplementary Fig. 5a, b; Supplementary Table 9). Importantly, the observed *APOE*-associated transcriptional differences were not driven by *SNCA*-triplication cases, but instead reflected shared molecular patterns across LBD samples stratified by *APOE* genotype (Supplementary Fig. 5a). The majority of DEGs were found in L5, with 76 upregulated and 203 downregulated genes enriched in this layer. Notably, the PD risk gene *PINK1* was upregulated in both L4 and L5, alongside other genes associated with synaptic and bioenergetic functions. The *CALM3* gene, encoding the calcium sensor calmodulin, was consistently upregulated, while the *HIPK2* gene, a kinase regulating transcription and apoptosis, was downregulated across all layers (Fig. 3c). Furthermore, pathway analysis revealed that upregulated DEGs were enriched in common pathways across GM layers, such as synaptic pathways, mitochondrial dysfunction, Parkinson’s signaling pathway and bioenergetic metabolism, while downregulated DEGs were enriched in myelination signaling pathway, stress response pathway, and lipid metabolism (Fig. 3d; Supplementary Table 10).

To assess whether these *APOE4*-related molecular alterations extended beyond LB+ spots, we also conducted DEG analyses in LBsur and LB-spots (Supplementary Fig. 5c, d; Supplementary Table 9). A subset of 56 upregulated and 124 downregulated genes were shared across LB annotation, suggesting widespread *APOE4*-related molecular alterations. Notably, 27 upregulated and 76 downregulated genes were uniquely altered in LB+ spots, many of which were enriched in pathways related to bioenergetic stress and transcriptional regulation (Supplementary Fig. 5e–g).

Collectively, these data indicate that *APOE4* exacerbates molecular dysfunction in LBD brains characterized by increased *SNCA* expression and pronounced layer-specific transcriptional alterations, particularly in L5, affecting synaptic and bioenergetic processes, as well as myelination and lipid metabolism.

### Synaptic and bioenergetic pathway dysregulation in L5 excitatory neurons of *APOE4* LBD brains

**Fig. 4:**
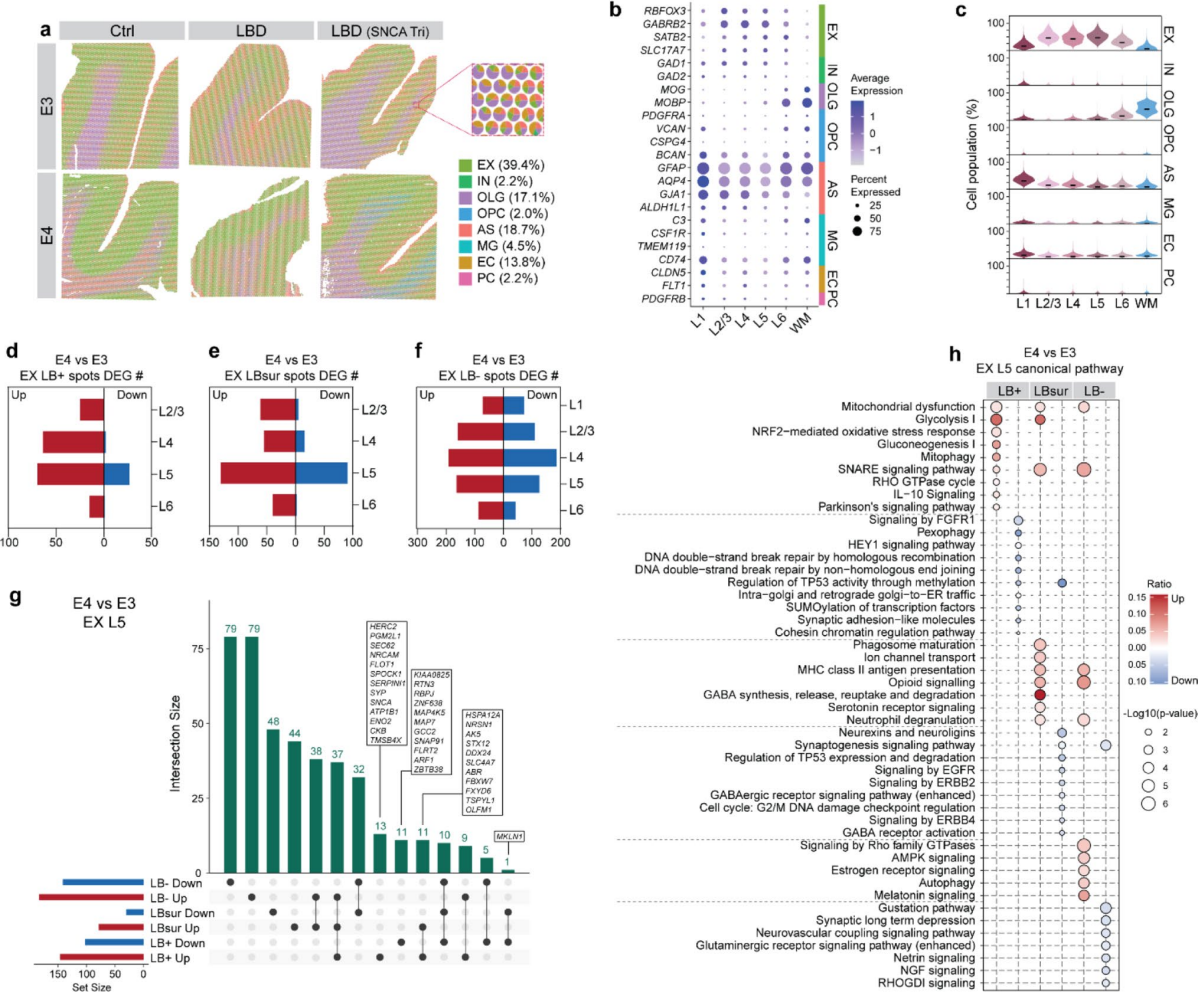
*APOE4* modifies cell-type specific alterations in response to LB pathology. **a** Spatial pie charts showing the cell-type composition at the spot level in E3 and E4 LBD brains, as well as Ctrl brains. Cell types include excitatory neurons (EX), inhibitory neurons (IN), oligodendrocytes (OLG), oligodendrocyte progenitor cells (OPC), astrocytes (AS), microglia (MG), endothelial cells (EC), and pericytes (PC). **b** Expression of cell-type marker genes across GM layers (L1–L6) and WM in LBD and control brains. **c** Violin plots showing the cell population across GM layers (L1–6) and WM. Bar graphs showing the number of DEGs in EX from LB+ (**d**), LBsur (**e**), and LB− (**f**) spots across GM layers (L1–L6) comparing E4 and E3 LBD brains. **g** Upset plot displaying annotated DEGs in EX from LB+, LBsur, and LB− spots in layer 5 (L5) comparing E4 and E3 LBD brains. **h** IPA canonical pathway analysis of DEGs enriched in EX from LB+, LBsur, and LB− spots in L5 comparing E4 and E3 LBD brains

To further uncover cell-type-specific transcriptional alterations associated with *APOE4* in LBD, we deconvoluted the spatial transcriptomics data using our previously published snRNA-seq dataset from human LBD brains [24]. Based on marker gene expression, we predicted cell population across GM layers and WM (Fig. 4a–c). Excitatory neurons (EX) constituted the largest population (39.4%) and were distributed across L2/3 to L6, while inhibitory neurons (IN, 2.2%) were predominantly enriched in L1. Astrocytes (AS, 17.1%) were most abundant in L1 but present in all GM layers and WM, and oligodendrocytes (OLG, 17.1%) were largely enriched in L6 and WM. Other cell types included microglia (MG, 4.5%), oligodendrocyte precursor cells (OPC, 2.0%), endothelial cells (EC, 13.8%), and pericytes (2.2%). Cell-type distributions were comparable between control and LBD brains across GM layers and WM (Supplementary Fig. 6a), regardless of LB annotations or *APOE* genotypes (Supplementary Fig. 6b). Consistent with our previous work [24], *SNCA* expression was predominantly found in EX, followed by OLG and MG (Supplementary Fig. 6c).

Given the comparable cell-type composition across conditions, we next asked whether glial cells are locally enriched in LB-associated regions independent of cortical layer or *APOE* genotype. Using deconvoluted cell-type percentage estimates from spatial transcriptomics, we observed no significant differences in microglial or astrocyte proportions between LB+ and LBsur spots (Supplementary Fig. 7a, b). To validate these findings histologically, we performed immunofluorescence co-staining in an independent cohort (Supplementary Table 11) using phosphorylated α-SYN to label LBs, together with Iba1 and GFAP to identify microglia and astrocytes, respectively. Glial signal intensity was quantified within concentric regions centered on LBs, including areas within 100 μm of LBs (approximating LB+ regions annotated in spatial transcriptomic slides) and surrounding regions 100–150 μm away (approximating LBsur regions). Consistent with the spatial transcriptomics analysis, microglial and astrocytic coverage did not differ significantly between these regions (Supplementary Fig. 7c–e). Together, these results indicate that local microglial and astrocyte abundance is largely unchanged in LB-associated regions, with no detectable enrichment in close proximity to LBs, in contrast to the well-described clustering of astrocytes and microglia around amyloid plaques. Nevertheless, glial cells may still respond to Lewy pathology through non-local, paracrine, or secreted inflammatory signaling mechanisms.

To determine the cell types driving *APOE4*-associated transcriptional changes near LB pathology, we performed cell-type-specific DEG analysis comparing E4 versus E3 LBD brains within LB+ and LBsur spots (Supplementary Table 12). The majority of DEGs were found in EX, followed by AS, across GM layers in both LB+ and LBsur spots (Fig. 4d, e; Supplementary Fig. 8a, b), whereas other cell types showed minimal DEG alterations (Supplementary Table 12). Notably, the most pronounced DEG enrichment occurred in EX of L5 in both LB+ and LBsur spots (Fig. 4d, e). To further distinguish LB-associated gene expression in EX, we analyzed DEGs in EX of LB+ and LBsur spots compared to LB− spots (Fig. 4d–g). In EX L5 of LB + spots, we identified 13 upregulated and 11 downregulated signature genes, with 11 upregulated and 1 downregulated gene commonly altered across both LB+ and LBsur spots (Fig. 4g). Pathway analysis of these DEGs revealed consistent upregulation of mitochondrial and synaptic dysfunction pathways in EX L5 across all LB annotations, while Parkinson’s signaling was uniquely enriched in EX L5 of LB+ spots (Fig. 4h; Supplementary Table 13).

To further explore the role of AS in L5, pathway analysis was performed with downregulated DEGs identified in L5 of LB+ and LBsur spots (Supplementary Fig. 8c). In both LB+ and LBsur spots, AS exhibited downregulation of Reelin signaling and immune-response pathways (Supplementary Fig. 8c), although bulk protein-level analyses showed no significant differences in Reelin pathway components between E3 and E4 LBD cases (Supplementary Fig. 4a–f). Other cell types showed limited DEGs in LB+ and LBsur spots but more substantial changes in L5 of LB− spots (Supplementary Fig. 8d–h; Supplementary Table 12).

These results identify excitatory neurons, particularly in L5, as a key cell type of the *APOE4*-associated bioenergetic and synaptic dysfunctions in LBD pathology, with additional alterations observed in other cell types, including astrocytes.

### Dysregulated cell–cell communication in GM and WM of *APOE4* LBD brains

**Fig. 5:**
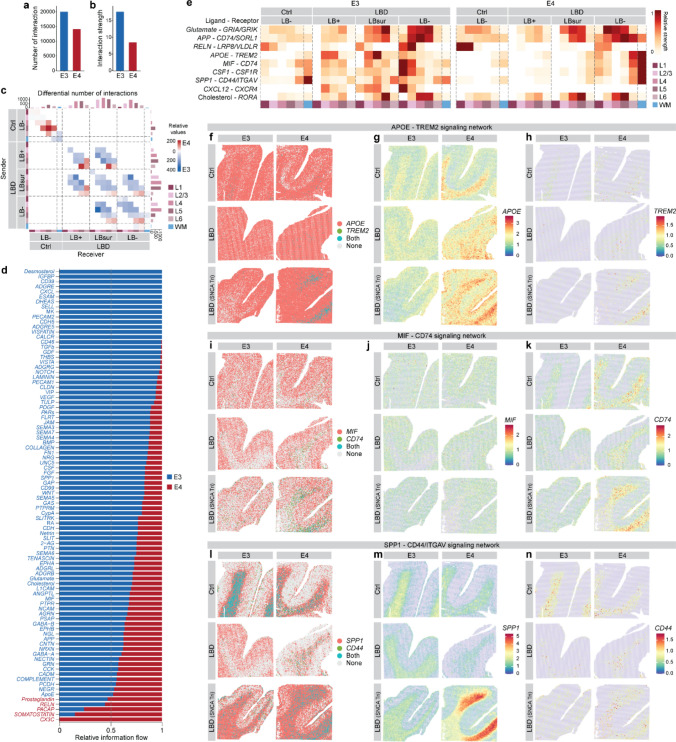
*APOE4* impairs cell–cell communication in LBD brains. Bar plots showing the total number of cell–cell communication (**a**) and interaction strength (**b**) in E3 (*blue*) and E4 (*red*) in Ctrl and LBD brains. **c** Heatmap illustrating the differential cell–cell communication interaction strength between LB+, LBsur, and LB− spots in E3 and E4 LBD brains, across GM layers (L1–6) and WM. Bar plots on the top and right sides display the sum of incoming and outgoing signaling, respectively. **d** Bar plots depicting signaling pathways with significant differences between E3 and E4 LBD brains, ranked by their information flow. Ligands for each signaling pathway are indicated on the left side of the bar plots. **e** Heatmap showing the relative strength of selected signaling pathways within LB+, LBsur, and LB− spots in E3 and E4 brains of Ctrl and LBD cases across GM layers (L1–6) and WM. Ligands and receptors for each signaling pathway are indicated on the left side of the heatmap. Spatial binary plots of ligand and receptor expression (**f**, **i**, **l**) and spatial gene expression plots of each ligand (**g**, **j**, **m**) and receptor (**h**, **k**, **n**) in selected immune-related signaling networks

To further investigate the contributions of potential changes in cell–cell interactions to *APOE4*-driven LBD pathogenesis, we assessed spot-based intercellular communication in E4 and E3 brains across GM layers and WM using CellChat [23]. Overall, E4 brains exhibited a reduced number and strength of cell–cell interactions compared to E3 brains (Fig. 5a–c). Specifically, multiple neuronal, immune-related, and lipid-related signaling pathways exhibited significantly altered interactions in E4 compared to E3 brains, with most signaling networks appearing weakened in E4 brains (Fig. 5d).

To better understand how these intercellular interaction changes relate to disease status (Ctrl, LBD) and LB annotations (LB+, LBsur, LB−), we analyzed key pathways across GM layers and WM within each *APOE* genotype, including the neuronal signaling (Glutamate—*GRIA/GRIK*, *APP—CD74/SORL1*, *RELN—LRP8/VLDLR*), immune-related signaling (*APOE—TREM2*, *MIF—CD74*, *CSF1—CSF1R*, *SPP1—CD44/ITGAV*, *CXCL12—CXCR4*), and lipid-related signaling (Cholesterol—*RORA*) (Fig. 5d, e). Neuronal and lipid signaling patterns were largely similar between E3 and E4 brains, with higher activity in LBsur and LB− regions compared to LB+ in LBD (Fig. 5e), consistent with reduced synaptic and Reelin signaling associated with neuronal vulnerability in LBD (Figs. 1, 2). In contrast, immune-related signaling showed striking differences: in E3 LBD brains, immune signaling was enriched across all LB annotations in both GM and WM, whereas in E4 LBD brains, immune signaling was primarily enriched in WM, with relatively little activation in GM—even in the presence of LBs—suggesting region-specific immune dysregulation in E4 brains (Fig. 5e). This WM-enriched immune activation in E4 LBD brains appeared to be driven by increased expression of ligands or receptors, as shown by ligand and receptor binary expression plots (Fig. 5f, i, l), ligand gene expression (Fig. 5g, j, m), and receptor gene expression (Fig. 5h, k, n). These ligands and receptors were primarily expressed by glial cells, particularly astrocytes and microglia.

**Fig. 6:**
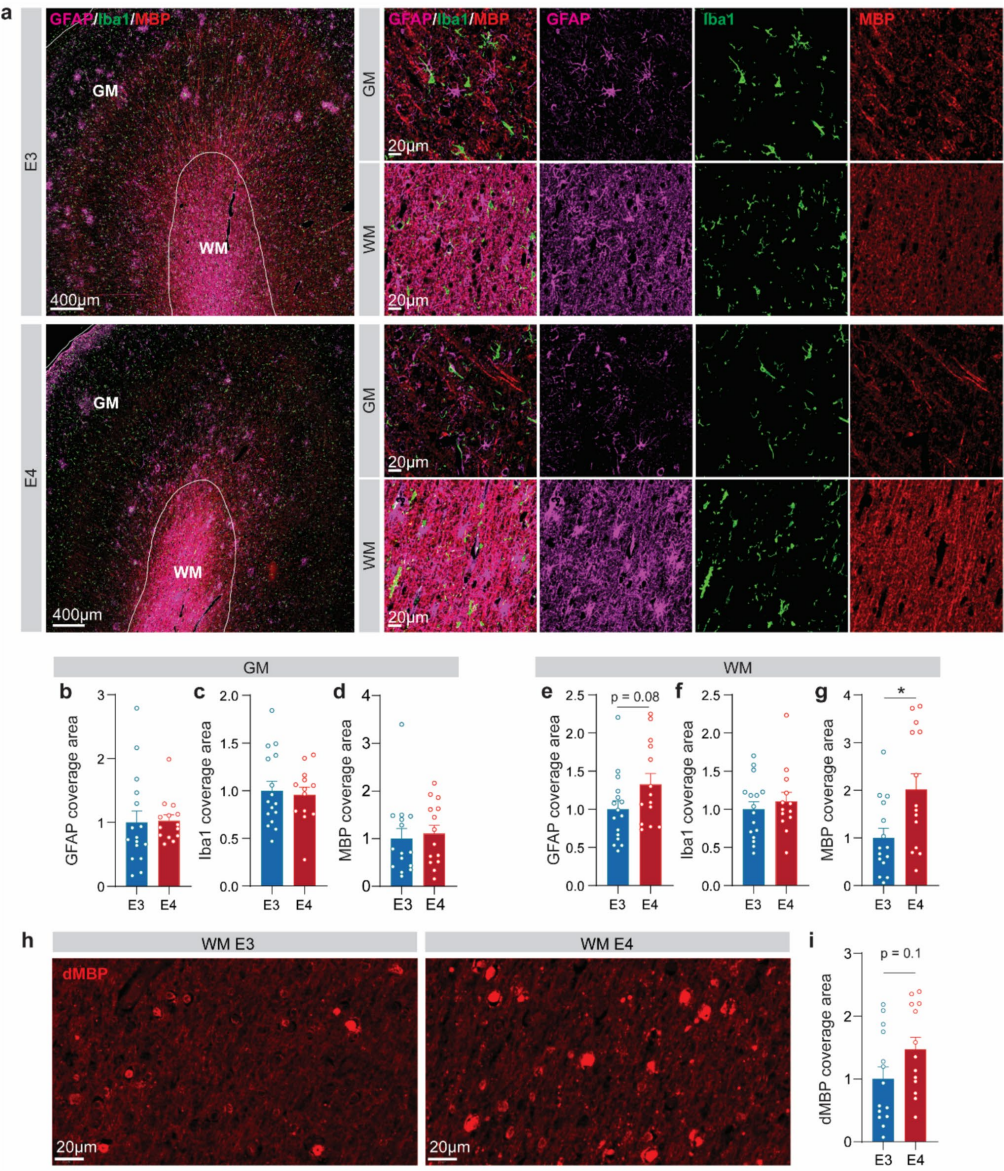
*APOE4* shows myelin accumulation and astrocyte responses in the white matter of LBD brains. Fluorescence staining images of the temporal cortex from human LBD brains showing GFAP (*magenta*), Iba1 (*green*), and MBP (*red*) (**a**). Quantification of GFAP (**b**, **e**), Iba1 (**c**, **f**), and MBP (**d**, **g**) coverage normalized to E3 in GM and WM of LBD brains. Scale bar: 400 μm (overview) or 20 μm (*inset*). Representative fluorescence images (**h**) and quantification (**i**) of dMBP coverage in the WM of E3 and E4 LBD brains. Scale bar: 20 μm. *N* = 16 E3 LBD brains and *N* = 13 E4 LBD brains. Data are presented as means ± SEM. Student’s *t* tests were used for statistical analyses. *p < 0.05

To determine whether this shift reflects gliosis and how it impacts WM, we performed immunostaining of temporal cortex sections spanning both GM and WM in E4 and E3 LBD brains (Fig. 6; Supplementary Fig. 9; Supplementary Table 11). Notably, GFAP+ reactive astrocyte coverage showed an increasing trend in WM of E4 LBD brains compared to E3 (*p* = 0.08), with no change in GM (Fig. 6a, b, e). In contrast, microglial coverage (Iba1) and the proportion of phagocytic microglia (CD68/Iba1) showed no significant differences between *APOE* genotypes in either region (Fig. 6a, c, f; Supplementary Fig. 9a–g). Interestingly, myelin basic protein (MBP) coverage was significantly increased in the WM of E4 LBD brains despite no change in the number of oligodendrocytes (Olig2⁺ cells) (Fig. 6a, d, g; Supplementary Fig. 9h–j). Similar findings have been reported in AD, where elevated MBP levels in *APOE4* carriers were accompanied by increased levels of degraded MBP (dMBP) [30]. To assess whether a comparable phenomenon occurs in LBD, we examined dMBP immunoreactivity in E3 and E4 LBD brains. Consistent with observations in AD, dMBP staining showed a trend toward increased coverage in the WM of E4 LBD brains compared with E3 brains (*p* = 0.10), although substantial inter-sample variability was observed (Fig. 6h, i). Together, these findings suggest that *APOE4* is associated with increased myelin debris accumulation and enhanced astrocytic responses in the WM of LBD brains.

To further investigate glial-specific dysfunction in WM at the transcriptomics level, we performed DEG analysis of deconvoluted glial cells (AS, MG, OLG, OPC) by comparing E4 versus E3 LBD brains (Supplementary Fig. 10a; Supplementary Table 14). Astrocytes and microglia exhibited upregulation of immune-response pathways, including neutrophil degranulation, MHC class II antigen presentation, macrophage classical activation signaling pathway, and interferon alpha/beta signaling (Supplementary Fig. 10b, c; Supplementary Table 15). Meanwhile, OLG and OPC showed upregulation of oxidative stress response, mitochondrial dysfunction, and myelination signaling pathway (Supplementary Fig. 10d, e; Supplementary Table 15).

Together, these results suggest that immune responses in E4 LBD brains are mainly concentrated in WM, likely driven by myelin debris buildup, while remaining limited in the GM, which may further contribute to LBD pathogenesis.

## Discussion

Our study provides a comprehensive, spatially and cell-type-resolved perspective on how *SNCA* and *APOE4* influence LBD pathogenesis. By integrating spatial transcriptomics with snRNA-seq-based deconvolution and validating our findings using postmortem human brain tissue and iPSC-derived organoid models, we demonstrate that both GM and WM are affected in LBD. We show that *SNCA* expression patterns are closely linked to the vulnerability of neurons that develop LBs, and that Reelin signaling is impaired in LBD. Furthermore, *APOE4* modulates the spatial molecular landscape of LBD by exacerbating neuronal vulnerability in GM and promoting dysregulated glial responses in WM, potentially in response to myelin debris accumulation. Our study highlights distinct, spatially defined molecular mechanisms that drive LBD pathogenesis (Supplementary Table 16).

Using human iPSC-derived cortical organoid models generated from patients with *SNCA* triplication, as well as postmortem human brain tissue from the temporal cortex, we previously demonstrated that *SNCA* is highly expressed in excitatory neurons, but not in inhibitory neurons [24]. In the present study, spatial transcriptomic profiling further reveals that *SNCA* expression in neurons is also layer-specific, with enrichment in deep cortical layers—particularly L5—where LBs are more frequently observed. These findings align with recent spatial transcriptomic analyses of the cingulate gyrus in brains from individuals with PD, PDD, and DLB [19]. Our data show that this layer-specific pattern of *SNCA* expression is consistent across control brains, LBD cases, and LBD cases with *SNCA* triplication. While *SNCA* expression levels are markedly elevated in *SNCA*-triplication cases, we also observe increased *SNCA* levels in sporadic LBD cases without triplication, suggesting that elevated *SNCA* expression—regardless of genetic cause—may contribute to neuronal vulnerability in LBD. The pathways disrupted between LBD and control brains, particularly those related to synaptic and bioenergetic function, mirror alterations associated with *SNCA* expression levels, further suggesting that the increased *SNCA* expression contributes to the LBD pathogenesis. Although the factors driving increased *SNCA* expression in sporadic LBD remain unclear, we found that *APOE4* carriers exhibit significantly higher levels of *SNCA* expression compared to non-carriers, and *APOE4* further exacerbates the excitatory neuron-specific transcriptional changes associated with synaptic and bioenergetic functions in LB pathology regions. This suggests that modulation of region-specific *SNCA* expression may be one mechanism through which *APOE4* increases LBD risk.

Reelin is a large, secreted glycoprotein that binds to several membrane receptors, including ApoER2, and plays a critical role in modulating synaptic plasticity and memory [1, 3]. Interestingly, the Reelin signaling pathway has been implicated in modulating the vulnerability of both excitatory and inhibitory neurons in AD. A recent study reported a significant reduction in the proportion of Reelin-expressing excitatory neurons in the entorhinal cortex (EC) of individuals with AD, as well as in amyloid-based animal models, suggesting a selective vulnerability of these neurons in AD pathogenesis [33]. In addition, a gain-of-function variant in the *RELN* gene—shown to enhance activation of its canonical downstream effector Dab1—was found to ameliorate AD pathology caused by *PSEN1* mutations [32], although further experimental validation is required. In our study, we found that Reelin signaling may also be involved in LBD pathogenesis and contribute to the neuronal vulnerability observed in this context. Impaired Reelin pathway activity appeared to be related to increased *SNCA* expression, as our *SNCA*-triplication organoids exhibited disrupted downstream Reelin signaling as well. However, the exact mechanisms through which Reelin contributes to α-SYN pathology remain to be elucidated. Although the Reelin receptor ApoER2 is also a receptor for APOE [46, 51], and prior studies have shown that APOE4 can impair Reelin signaling by disrupting ApoER2 recycling [12], our data do not support a further modulation of Reelin signaling by APOE4 at the bulk protein level in LBD. These findings indicate that Reelin pathway alterations in LBD are likely disease-intrinsic rather than primarily driven by *APOE* genotype.

Another important molecular phenotype we identified when comparing LBD and control brains is alterations in WM, including downregulation of myelination-related pathways and associated lipid dysregulation, both of which are essential for proper myelin maintenance. These findings suggest that WM damage is a feature of LBD and may be linked to neuronal injury caused by α-SYN accumulation [11]. Notably, we also observed an increased accumulation of myelin debris in *APOE4* carriers with LBD compared to non-carriers. This phenotype is consistent with our recent findings in AD brains, where myelin debris accumulation was similarly enriched in *APOE4* carriers [30]. These results point to a potential defect in myelin clearance in *APOE4* carriers, possibly involving mechanisms shared between AD and LBD [4, 30]. Interestingly, our analysis of intercellular signaling networks revealed that *APOE4* LBD brains exhibit reduced overall cell–cell communication in the GM, where LBs are primarily located, alongside selectively increased immune-related signaling in WM. This enhanced immune signaling may be linked to the observed myelin debris accumulation in *APOE4* carriers, suggesting that *APOE4*-associated gliosis contributes to a chronically inflamed and metabolically stressed WM environment [29, 35]. This spatial uncoupling of glial responses—characterized by suppressed communication in GM and hyperactive immune signaling in WM—may impair interregional communication and exacerbate cortical vulnerability by disrupting long-range axonal integrity. Together, these findings underscore the role of dysregulated glial activation and region-specific cellular responses in further mediating the increased vulnerability associated with *APOE4* in LBD pathogenesis.

Prior spatial transcriptomic studies have identified synaptic and mitochondrial dysfunction in cortical neurons associated with Lewy pathology, with particular vulnerability of deep-layer excitatory neurons [19]. Our findings are highly consistent with these observations, as we similarly detect pronounced synaptic and bioenergetic pathway dysregulation in L5 associated with Lewy bodies. Importantly, our study extends the prior work by integrating glial responses, white matter myelin disruption, genetic risk conferred by *APOE4*, and multimodal validation, providing a more comprehensive spatial and cellular framework for understanding LBD pathology.

Altogether, our study highlights the molecular landscape of LBD and reveals how *SNCA* expression and *APOE4* may contribute to neuronal vulnerability and dysregulated glial responses in LBD pathogenesis. Our findings underscore the importance of spatial context and cell-type specificity in understanding the mechanisms underlying neurodegenerative diseases. Future studies will be essential to delineate the causal sequence of these molecular events and to explore the translational potential of targeting *SNCA* and *APOE4* to modify the progression of LBD.

## Supplementary Information

Below is the link to the electronic supplementary material.

Supplementary Table 9 (XLSX 9719 KB)

Supplementary Table 10 (XLSX 11 KB)

Supplementary Table 11 (XLSX 16 KB)

Supplementary Table 12 (XLSX 160 KB)

Supplementary Table 13 (XLSX 67 KB)

Supplementary Table 14 (XLSX 19 KB)

Supplementary Table 15 (XLSX 10 KB)

Supplementary Table 16 (XLSX 57 KB)

Supplementary Figures (DOCX 10 KB)

Supplementary Table 1 (XLSX 363 KB)

Supplementary Table 2 (XLSX 167 KB)

Supplementary Table 3 (XLSX 10 KB)

Supplementary Table 4 (XLSX 185 KB)

Supplementary Table 5 (XLSX 101 KB)

Supplementary Table 6 (XLSX 35 KB)

Supplementary Table 7 (XLSX 43 KB)

Supplementary Table 8 (XLSX 10 KB)

## Data Availability

Raw and processed spatial transcriptomics data are deposited in the National Center for Biotechnology Information’s Gene Expression Omnibus under accession numbers GSE293896. The codes used for the data analysis are available on GitHub (https://github.com/ZhaoNaLab/LBD-Spatial.git) and Zenodo (10.5281/zenodo.15336929).
