# Supplementary material for: Spatially resolved molecular signatures of Lewy body dementia
Source: Acta Neuropathol. 2026 Jan 26;151(1):10. doi: 10.1007/s00401-026-02981-z (PMC12835040; doi:10.1007/s00401-026-02981-z)
Supplement: Supplementary file 9 — Supplementary Figures (DOCX 10 KB) [file 401_2026_2981_MOESM9_ESM.docx]

**Supplementary Information**

**Spatially resolved molecular signatures of Lewy body dementia**

*Jin et al*.

**Inventory**

**Supplementary Figures**

Supplementary Fig. 1. H&E staining, cortical layer annotation, and Lewy body distribution in spatial transcriptomics slides.

Supplementary Fig. 2. Quality control metrics for spatial transcriptomics data.

Supplementary Fig. 3. UMAP visualization of spatial transcriptomics spots before and after data integration.

Supplementary Fig. 4. Analysis of Reelin signaling by western blot in *APOE3* and *APOE4* LBD brains.

Supplementary Fig. 5. *APOE4* modulates the transcriptional signatures in LBD brains.

Supplementary Fig. 6. Cell population changes related to disease status, *APOE* genotype, and LB annotation.

Supplementary Fig. 7. Microglial and astrocytic distribution relative to Lewy bodies.

Supplementary Fig. 8. *APOE4*-related cell-type specific changes in response to LB pathology.

Supplementary Fig. 9. Microglia and oligodendrocyte lineage cell densities in LBD brains with different *APOE* genotypes.

Supplementary Fig. 10. *APOE4* modifies glial cell-type specific responses in WM of LBD brains.

**Supplementary Tables**

Supplementary Table 1. Patient characteristics of postmortem human brain for spatial transcriptomics.

Supplementary Table 2. Spatial transcriptomic spot coverage across samples, regions, and analytical categories.

Supplementary Table 3. DEG list: LBD vs Ctrl across GM layers and WM.

Supplementary Table 4. Pathway list: LBD vs Ctrl across GM layers and WM.

Supplementary Table 5. DEG and pathway list: LB+ vs LB- spots & LBsur vs LB- spots in GM.

Supplementary Table 6. Patient characteristics of the postmortem human brains for western blotting validation (Control vs LBD).

Supplementary Table 7. DEG and pathway list: *SNCA* high vs *SNCA* low in LB+ and LBsur spots in GM.

Supplementary Table 8. Patient characteristics of the postmortem human brains for western blotting (LBD E3 vs E4).

Supplementary Table 9. DEG list: E4 vs E3 in LB+, LBsur, and LB- spots across GM layers.

Supplementary Table 10. Pathway list: E4 vs E3 in LB+, LBsur, and LB- spots across GM layers.

Supplementary Table 11. Patient characteristics of the postmortem human brains for staining validation.

Supplementary Table 12. DEG list: E4 vs E3 in deconvoluted cell types in LB+, LBsur and LB- spots across GM layers.

Supplementary Table 13. Pathway list: E4 vs E3 in deconvoluted cell types in LB+, LBsur and LB- spots across GM layers.

Supplementary Table 14. DEG list: E4 vs E3 in deconvoluted cell types in WM.

Supplementary Table 15. Pathway list: E4 vs E3 in deconvoluted cell types in WM.

Supplementary Table 16. Summary of main findings of this study.

**Supplementary Figures and Legends**


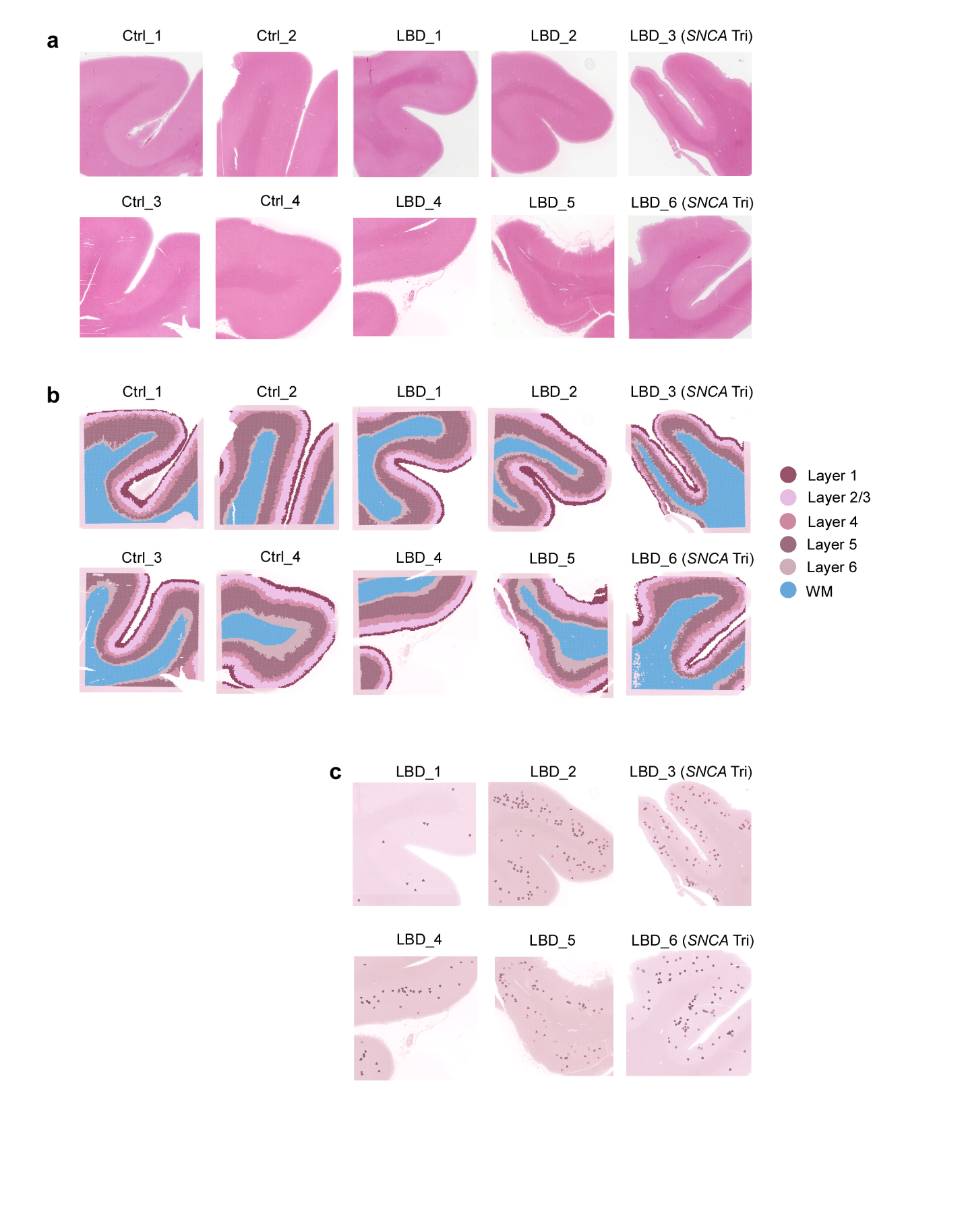


**Supplementary Fig. 1. H&E staining, cortical layer annotation, and Lewy body distribution in spatial transcriptomics slides.**

**(a)** H&E-stained temporal cortex sections from control (Ctrl_1-4) and LBD (LBD_1-6) cases used for spatial transcriptomics. LBD_3 and LBD_6 carry *SNCA* gene triplication (*SNCA* Tri).

**(b)** Spatial transcriptomics-based annotation of cortical layers (L1-L6) and white matter (WM) in the same sections.

**(c)** Lewy body annotation and distribution in LBD cases.


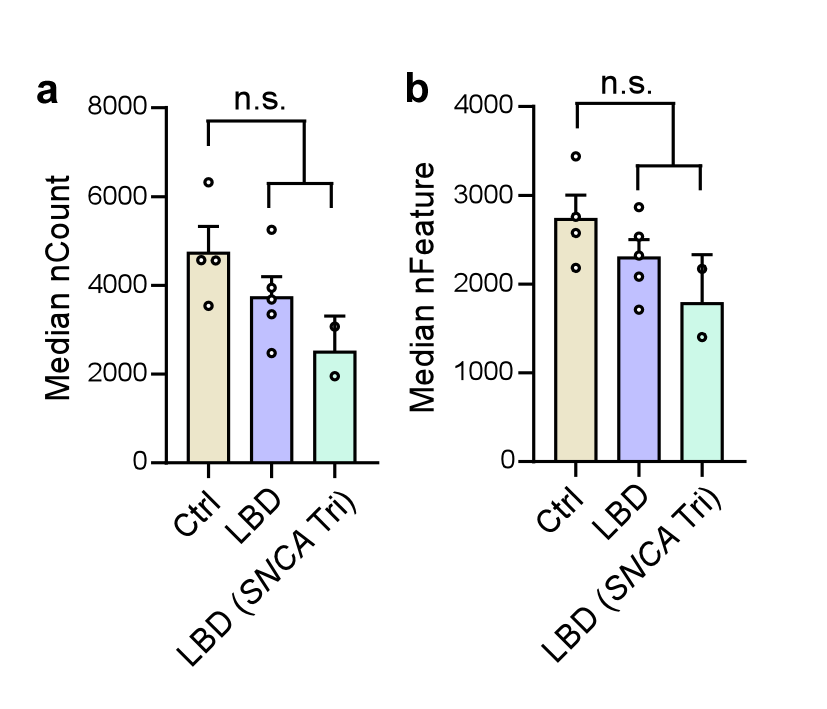


**Supplementary Fig. 2. Quality control metrics for spatial transcriptomics data.**

**(a)** Median UMI counts per spatial spot (nCount) for each sample across groups.

**(b)** Median number of detected genes per spatial spot (nFeature) for each sample across groups.

Data are presented as means ± SEM. Each dot represents one sample. Student's t-tests were used for statistical analyses. n.s., not significant.

**
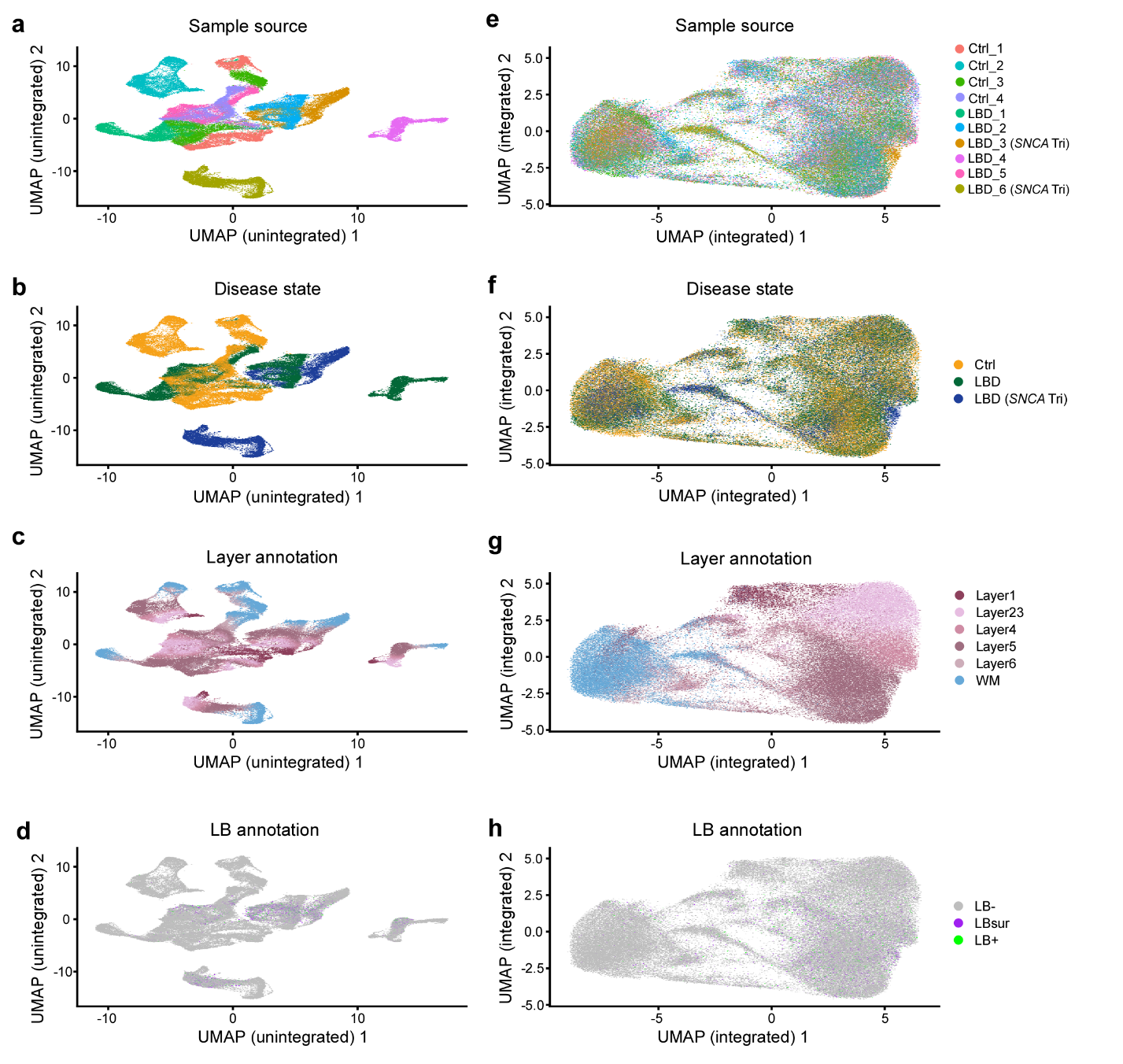
**

**Supplementary Fig. 3. UMAP visualization of spatial transcriptomics spots before and after data integration.**

**(a-d)** UMAP projections of unintegrated spatial transcriptomics spots showing sample source (a), disease state (b), cortical layer annotation (c), and Lewy body (LB) annotation (d).

**(e-h)** UMAP projections of the same spots after integration showing sample source (e), disease state (f), cortical layer annotation (g), and LB annotation (h).


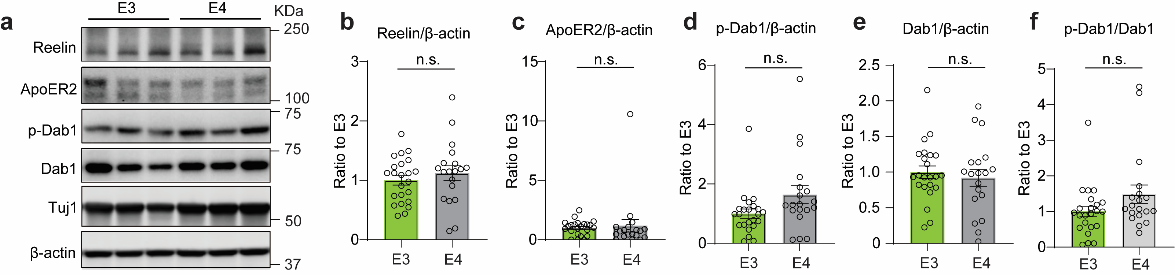


**Supplementary Fig. 4. Analysis of Reelin signaling by western blot in *APOE3* and *APOE4* LBD brains.**

**(a)** Representative western blot gel images.

**(b-f)** Quantification of Reelin (b), ApoER2 (c), p-Dab1 (d), Dab1 (e) levels, and p-Dab1/Dab1 ratio (f) in TBSX fractions. Results were normalized to β-actin levels. Human brains: N = 23 samples for E3 group, N = 19 samples for E4 group; each dot represents an individual case. Data are presented as mean ± SEM. Statistical analyses were performed using Student's t-test. n.s., not significant.

**
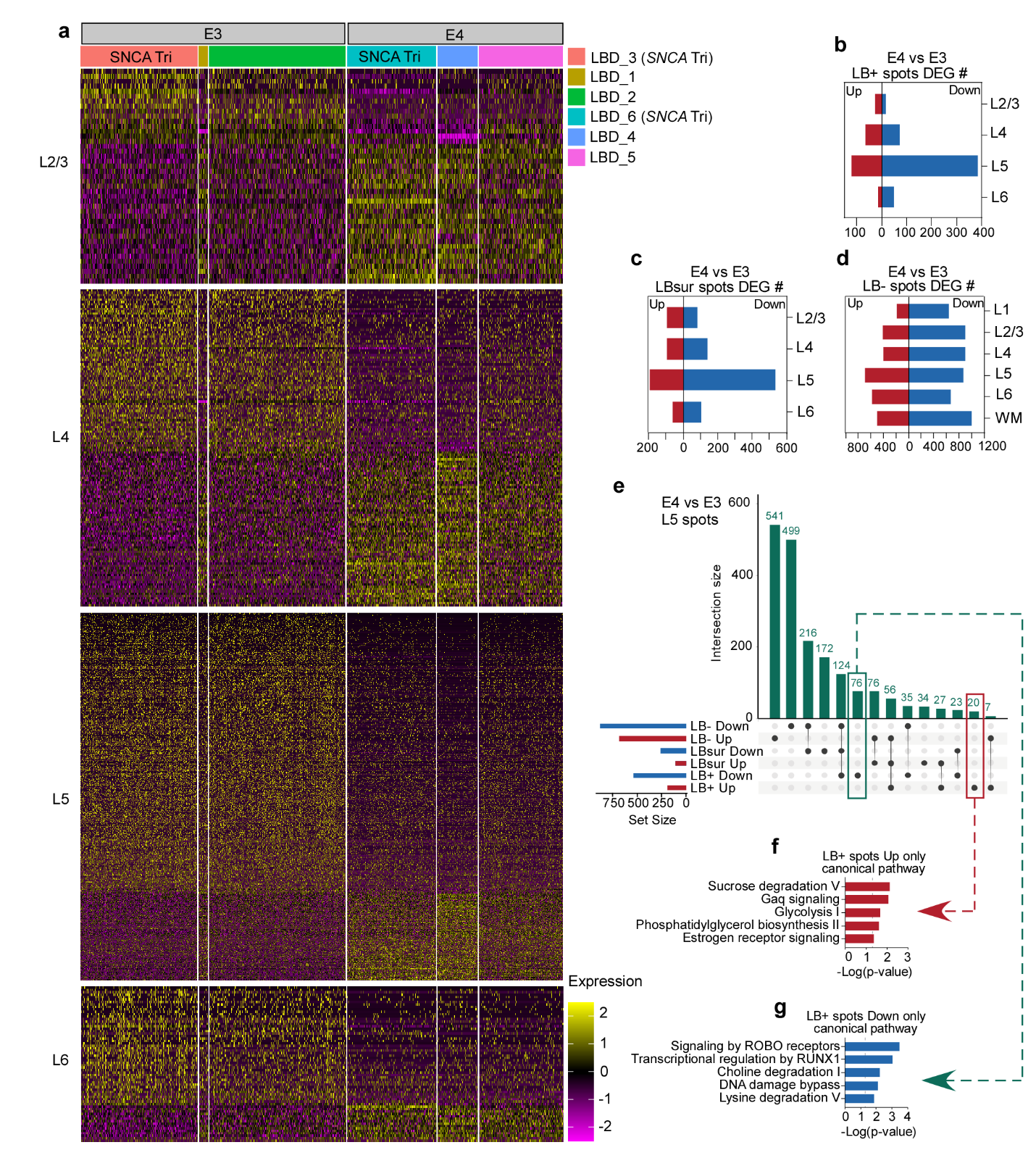
**

**Supplementary Fig. 5. *APOE4* modulates the transcriptional signatures in LBD brains.**

**(a)** Heatmap showing layer-specific DEGs in LB+ spots for individual E3 and E4 LBD brains.

**(b-d)** Bar graphs showing the number of DEGs in LB+ spots (b), LBsur spots (c), and LB- spots (d) across GM layers (L1-6), comparing E4 and E3 LBD brains.

**(e-g)** Upset plot showing annotated DEGs in LB+, LBsur, and LB- spots in L5, comparing E4 and E3 LBD brains or Ctrl brains (e), with IPA canonical pathway analysis of upregulated DEGs in LB+ spots of E4 LBD brains only (f) and downregulated DEGs in LB+ spots of E4 LBD brains only (g).


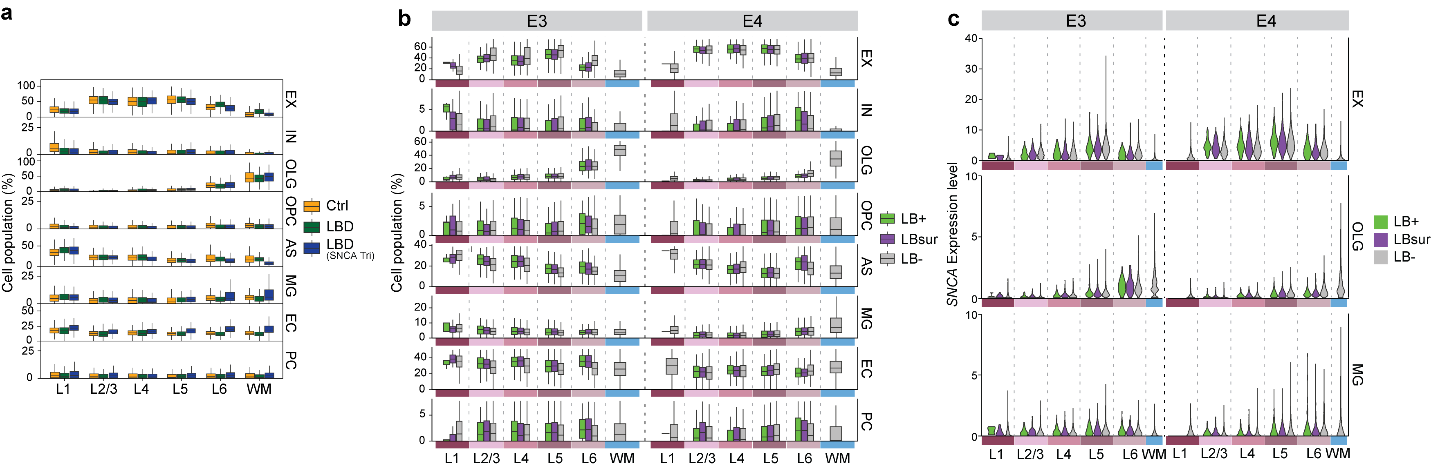


**Supplementary Fig. 6. Cell population changes related to disease status, *APOE* genotype, and LB annotation.**

**(a)** Box plots showing the cell population across GM layers (L1-6) and WM, grouped by disease status in Ctrl and LBD brains.

**(b, c)** Box plots showing the percentage of each cell type (b) and violin plots displaying *SNCA* gene expression (c) in LB+, LBsur, and LB- spots across GM layers (L1-6) and WM in LBD brains.


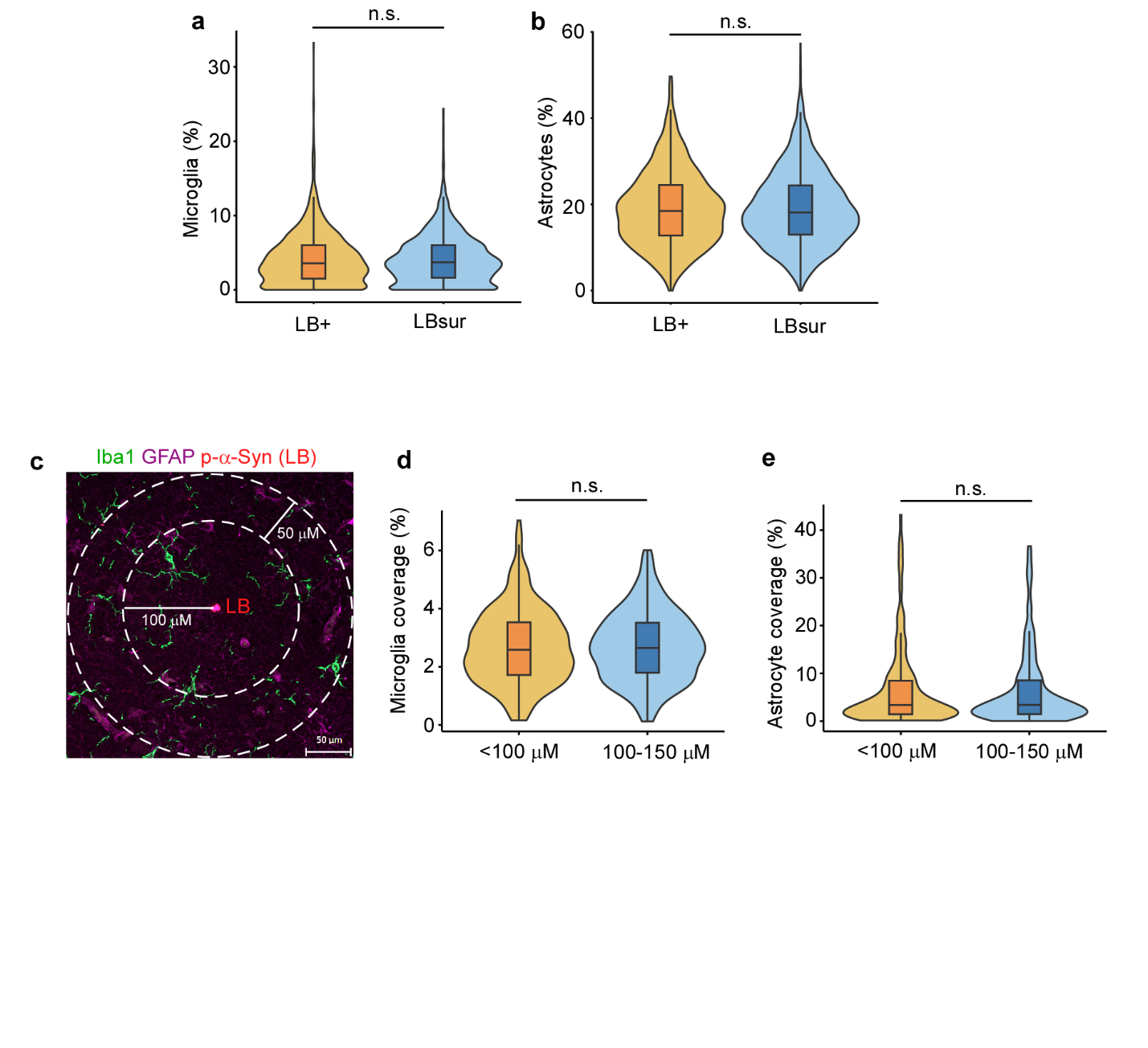


**Supplementary Fig. 7. Microglial and astrocytic distribution relative to Lewy bodies.**

**(a, b)** Violin plots showing the relative proportions of microglia (a) and astrocytes (b) in Lewy body-positive (LB+) and LB surrounding (LBsur) spatial transcriptomics spots.

**(c)** Representative immunofluorescence image showing Iba1⁺ microglia (green), GFAP⁺ astrocytes (magenta), and phosphorylated α-synuclein-positive LBs (p-α-Syn; red). Concentric regions indicate areas within 100 μm of LBs and surrounding regions (100-150 μm).

**(d, e)** Violin plots showing microglial (d) and astrocyte (e) coverage within regions located <100 μm or 100-150 μm from LBs.

Boxes indicate median and interquartile range; whiskers denote data spread. Statistical analyses were performed using Student's t-test. n.s., not significant.


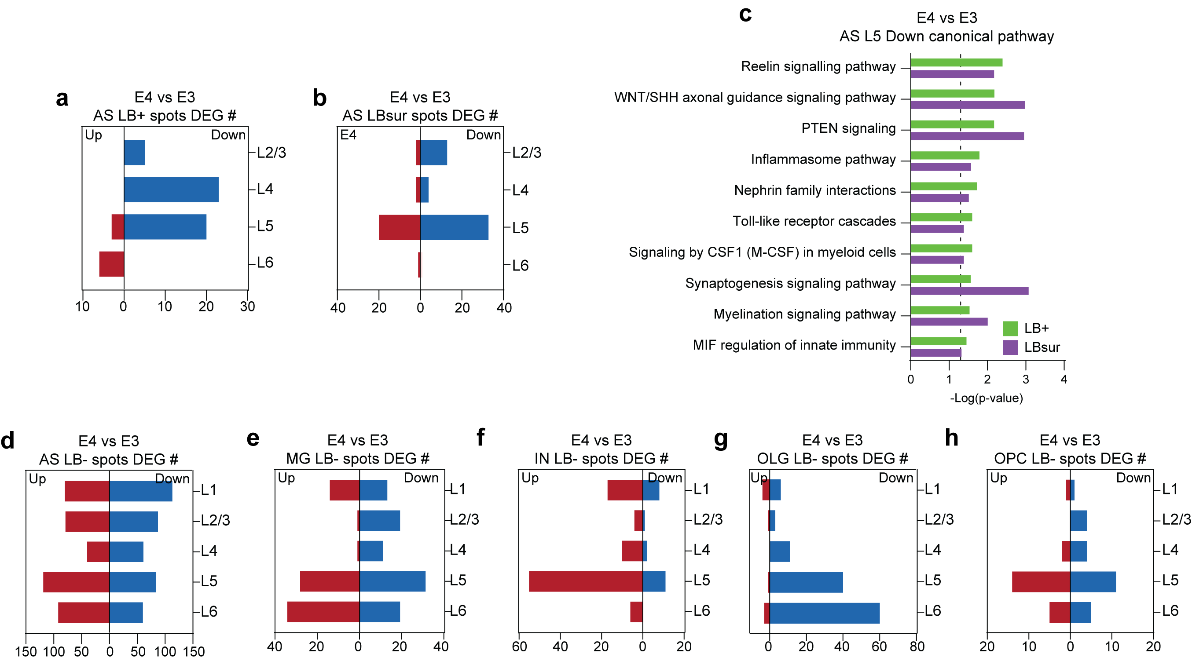


**Supplementary Fig. 8. *APOE4*-related cell-type specific changes in reponse to LB pathology.**

**(a, b)** Bar graphs showing the number of DEGs in AS of LB+ (a) and LBsur (b) spots across GM layers (L1-6), comparing E4 and E3 LBD brains.

**(c)** Common IPA canonical pathway analysis of downregulated DEGs enriched in astrocytes (AS) of LB+ and LBsur spots, comparing E4 and E3 LBD brains.

**(d-h)** Bar graphs showing the number of DEGs in AS (d), microglia (MG) (e), inhibitory neurons (IN) (f), oligodendrocytes (OLG) (g), and oligodendrocyte precursor cells (OPC) (h) in LB- spots across GM layers (L1-6), comparing E4 and E3 LBD brains.


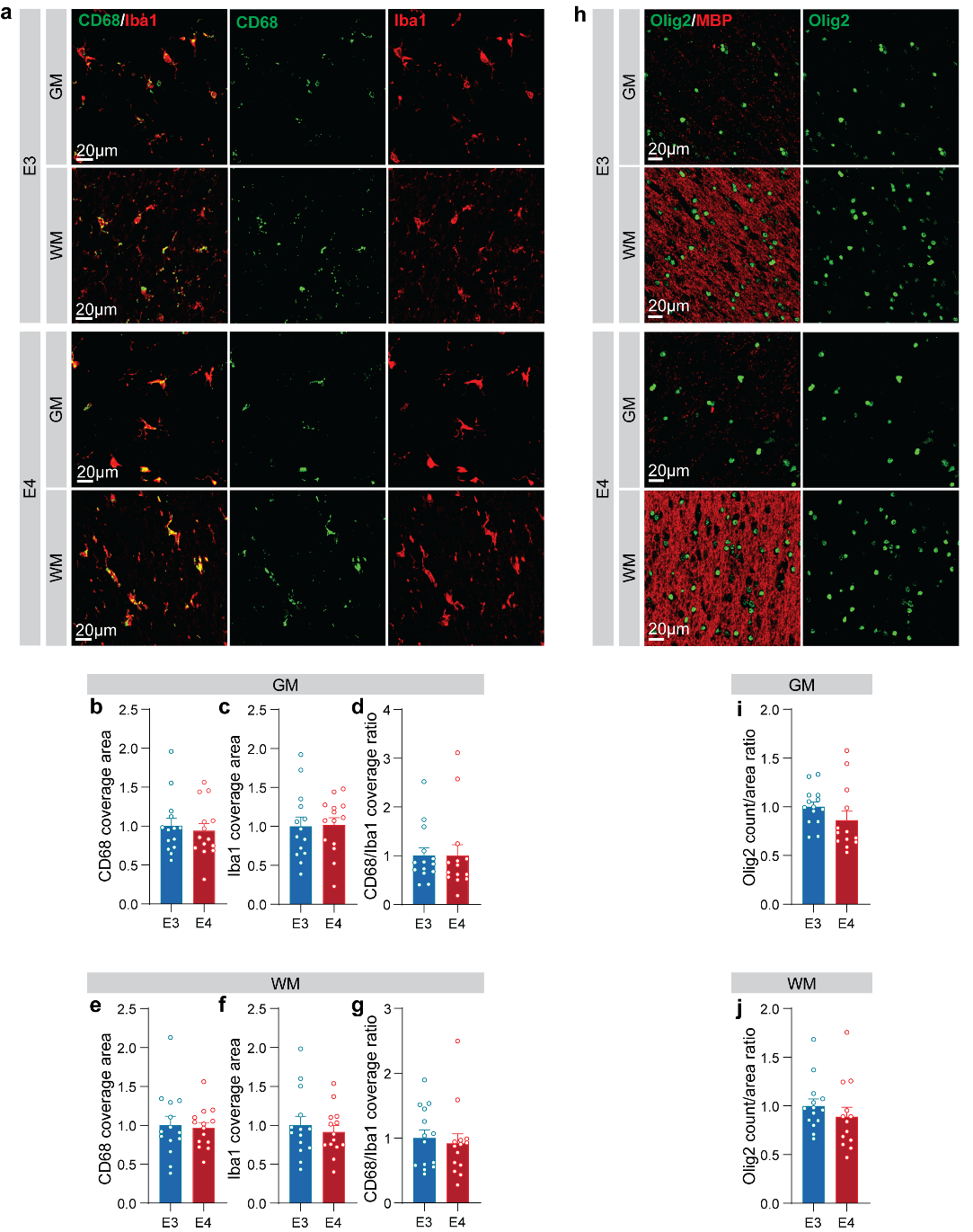


**Supplementary Fig. 9. Microglia and oligodendrocyte lineage cell densities in LBD brains with different *APOE* genotypes.**

**(a-g)** Fluorescence staining images of human LBD brains with CD68 (green) and Iba1 (red). Quantification of CD68 (b, e) and Iba1 (c, f) coverage, and the CD68 to Iba1 ratio (d, g) normalized to E3 in GM and WM.

**(h-j)** Fluorescence staining images of human LBD brains with Olig2 (green) and MBP (red). Quantification of Olig2 count per area (i, j) normalized to E3 in GM and WM.

Scale bars: 400 μm (overview) or 20 μm (insets). N = 14 E3 LBD brains and N = 14 E4 LBD brains. Data are presented as means ± SEM. Student's t-tests were used for statistical analyses.


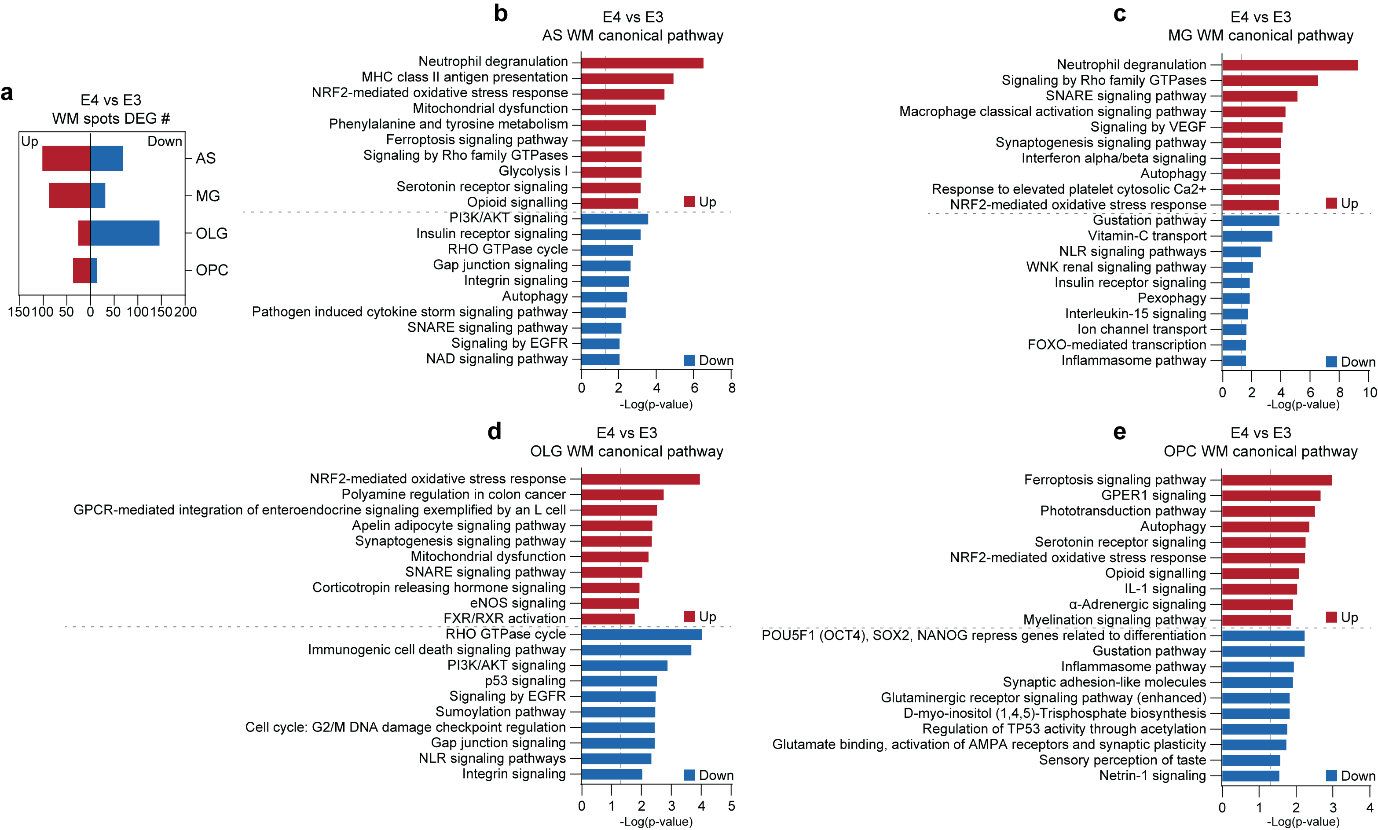


**Supplementary Fig. 10. *APOE4* modifies glial cell-type specific responses in WM of LBD brains.**

**(a)** Bar graph showing the number of DEGs in AS, MG, OLG, and OPC in WM, comparing E4 and E3 LBD brains.

**(b-e)** IPA canonical pathway analysis of DEGs enriched in AS (b), MG (c), OLG (d), and OPC (e) in WM, comparing E4 and E3 LBD brains.
